# Interval-valued distributed preference relation and its application to group decision making

**DOI:** 10.1371/journal.pone.0198393

**Published:** 2018-06-11

**Authors:** Yin Liu, Chao Fu, Min Xue, Wenjun Chang, Shanlin Yang

**Affiliations:** 1 School of Management, Hefei University of Technology, Hefei, Hefei, Anhui, P.R. China; 2 Key Laboratory of Process Optimization and Intelligent Decision-making, Ministry of Education, Hefei, Anhui, P.R. China; Southwest University, CHINA

## Abstract

As an important way to help express the preference relation between alternatives, distributed preference relation (DPR) can represent the preferred, non-preferred, indifferent, and uncertain degrees of one alternative over another simultaneously. DPR, however, is unavailable in some situations where a decision maker cannot provide the precise degrees of one alternative over another due to lack of knowledge, experience, and data. In this paper, to address this issue, we propose interval-valued DPR (IDPR) and present its properties of validity and normalization. Through constructing two optimization models, an IDPR matrix is transformed into a score matrix to facilitate the comparison between any two alternatives. The properties of the score matrix are analyzed. To guarantee the rationality of the comparisons between alternatives derived from the score matrix, the additive consistency of the score matrix is developed. In terms of these, IDPR is applied to model and solve multiple criteria group decision making (MCGDM) problem. Particularly, the relationship between the parameters for the consistency of the score matrix associated with each decision maker and those for the consistency of the score matrix associated with the group of decision makers is analyzed. A manager selection problem is investigated to demonstrate the application of IDPRs to MCGDM problems.

## Introduction

With the development of emerging information technologies, such as Cloud Computing, Big Data, and Internet of Things, a large amount of information has been accumulated [1,2]. The accumulated information provides important foundations for handling complex problems. More importantly, real problems are usually required to be analyzed in a changing environment. All these indicate that it may be difficult to make rational decisions by depending on individual capabilities. Integrating the perceived and understanding capabilities of group to analyze real problems [3], becomes a trend in the current era. Many practical problems in different fields have been analyzed with the aid of collective intelligence of group, such as human resource management [4,5], the selection of strategic and green suppliers [6,7], the selection of project and portfolio management information system [8], project manager selection [9], and the evaluation of strategic emerging industries [10], portfolio investing [11].

To facilitate analyzing problems like what has been presented above, many group decision making methods have been developed in the uncertain context. In these methods, there are generally two types of ways to help decision makers express preferences. One is to evaluate alternatives solely and the other is to compare alternatives in pairs. In the first type of way, many different styles of uncertain expressions have been proposed to characterize the preferences of decision makers, such as fuzzy set [12–16], fuzzy linguistic term set [17,18], and belief distribution [19–22]. Also, there have been many other studies on how to express the preferences of decision makers for comparing alternatives in pairs. Among these studies, representative pairwise preferences include multiplicative preference relation (MPR) [23,24], fuzzy preference relation (FPR) [25–27], intuitionistic fuzzy preference relation (IFPR) [28,29], linguistic preference relation (LPR) [30,31], and distributed preference relation (DPR) [32]. When information is sufficient and decision makers are capable of handling the information available and using it to make decisions, they may prefer to evaluate alternatives solely. If the two conditions cannot be satisfied, decision makers may wish to make pairwise comparison among alternatives. Not only that, it is convenient and simple for decision makers to provide pairwise comparison among alternatives [32,33]. Both the sole evaluation of alternatives and the pairwise comparison among alternatives are helpful for aiding decision makers in solving complex problems in the uncertain environment, although their respective studies are different in many perspectives, such as preference collection, preference requirements, and preference processing to generate solutions. Considering these differences, we focus on group decision making with pairwise comparisons in this paper.

When facing practical problems, such as personnel selection in enterprise, decision makers usually compare candidates on some criteria by considering multiple perspectives or information from different sources. If MPR, IFPR, and LPR are used to express the preferences of decision makers in the process of modeling and solving the problems, decision makers need to combine different perspectives or information to provide pairwise preferences on each criterion. However, original information can be retained when decision makers express their preferences as DPRs because the preferred, non-preferred, indifferent, and uncertain degrees of one alternative over another are simultaneously reflected in DPR [32]. The DPRs seem to be more complex than MPRs, IFPRs, and LPRs in general styles. Their complexity is to relieve the burden on decision makers to aggregate the information from different sources and characterize the real preferences of decision makers. When only the preferred degrees, the non-preferred degrees, or the indifferent degrees are required by decision makers to reflect their preferences, the complexity of DPRs will decrease significantly.

Although DPR considers multiple possibilities of one alternative over another, it may be inapplicable in some situations. For example, when clinicians compare two radiologists’ capabilities of diagnosing thyroid cancers, they may prefer to provide interval-valued rather than precise degree to which one radiologist is preferred to, non-preferred to, or indifferent to another. Although the clinicians have read a large number of examination reports provided by the two radiologists, they give the comparison between the two radiologists by their impression on them rather than precise statistics for their examination reports. In addition, diagnoses of thyroid cancers are also influenced by some controllable and uncontrollable factors, which increase the uncertainty as to the true diagnoses of thyroid cancers. When facing practical problems similar to the comparison between two radiologists, decision makers may wish to express interval-valued preferred, non-preferred, indifferent, and uncertain degrees of one alternative over another. This brings a challenge of extending DPRs to model and analyze group decision making problems in such situations. Similar to DPRs, the complexity of the extension of DPRs is to reflect the real preferences of decision makers. The complexity will also decrease significantly when only the interval-valued preferred degrees, the interval-valued non-preferred degrees, or the interval-valued indifferent degrees are required by decision makers.

To address the challenge, in this paper, we propose the concept of interval-valued distributed preference relation (IDPR) which aims to address the situations where a decision maker cannot provide the precise degrees of one alternative over another due to lack of knowledge, experience, and data. Because directly comparing two alternatives from IDPRs is not easy, an IDPR matrix is transformed into a score matrix through constructing two optimization models for any two alternatives. The properties of the score matrix and its application to compare alternatives are presented. To guarantee the rationality of the comparisons between alternatives from the score matrix, the additive consistency of the score matrix is developed and theoretically analyzed. With the consideration of the transformation from an IDPR matrix to its score matrix, the consistency of the score matrix, and the comparison between alternatives from the score matrix, IDPRs are used to model and analyze multiple criteria group decision making (MCGDM) problems. In particular, the relationship between the parameters for the consistency of the score matrix associated with each decision maker and those for the consistency of the score matrix associated with the group of decision makers is analyzed.

The rest of this paper is organized as follows. In Preliminaries, we briefly review some basic concepts of DPR. After that, we propose the concept of IDPR and present how to compare alternatives through the score intervals of IDPRs and how to guarantee the consistency of the score intervals of IDPRs. IDPRs are used to model and analyze MCGDM problems. In Illustrative example, a manager selection problem is investigated to demonstrate the application of IDPR to MCGDM. Finally, the paper is concluded in Conclusions.

## Preliminaries

In this section, we present basic concepts of DPR and its relevant consistency.

### DPR

Given a set of alternatives *X* = {*x*_1_, *x*_2_, …, *x*_*M*_}, the relationship between alternatives *x*_*i*_ and *x*_*j*_ can be profiled by a DPR.

**Definition 1.** [32] Let *X* = {*x*_1_,*x*_2_,…,*x*_*M*_} be a set of alternatives. Suppose that a set of grades *Ω* = {*H*_1_,*H*_2_,…,*H*_*N*_} with an odd number *N* is used to compare any two alternatives *x*_*i*_ and *x*_*j*_, where *H*_(*N*+1)/2_ stands for an indifferent grade, *H*_(*N*+1)/2+1_,…,*H*_*N*_ for the grades with increasing preferred intensity, and *H*_1_,…,*H*_(*N*+1)/2−1_ for the grades with decreasing non-preferred intensity. Then the comparison between alternatives *x*_*i*_ and *x*_*j*_ is profiled by a DPR, which is represented by *d*_*ij*_ = {(*H*_*n*_,*m*_*ij*_(*H*_*n*_)),*n* = 1,…,*N*; (*Ω*,*m*_*ij*_(*Ω*))}, where *m*_*ij*_(*H*_*n*_) denotes the belief degree assessed to grade *H*_*n*_ and *m*_*ij*_(*Ω*) denotes the belief degree of global ignorance between *x*_*i*_ and *x*_*j*_. The DPR satisfies:

$$0\leq m_{ij}(H_{n})\leq 1,\;0\leq m_{ij}(\Omega )\leq 1,\;\text{for}\;n\in \{1,\;\ldots ,N\},$$ (1)

$$\sum_{n=1}^{N}m_{ij}(H_{n})+m_{ij}(\Omega )=1,$$ (2)

$$m_{ij}(H_{n})=m_{ji}(H_{N}{}_{-n+1}),\;\text{for}\;n\in \{1,\;\ldots ,N\}$$ (3)

$$m_{ij}(\Omega )=m_{ji}(\Omega ),$$ (4)

and

$$m_{ii}(H_{(N+1)/2})=1.$$ (5)

Definition 1 shows that the preferred, non-preferred, indifferent, and uncertain degrees of alternative *x*_*i*_ over *x*_*j*_ are characterized by the DPR *d*_*ij*_. When DPRs between any two alternatives are obtained, a DPR matrix *D* = (*d*_*ij*_)_*M*×*M*_ is formed. It is clear that *D* ⊂ *X* × *X*. To illustrate the concept of a DPR, we present the following example.

**Example 1.** Suppose that *Ω* = {*H*_1_,*H*_2_,…,*H*_11_} = {absolutely inferior, much more inferior, more inferior, moderately inferior, marginally inferior, indifferent, marginally superior, moderately superior, more superior, much more superior, absolutely superior} denotes a set of grades used to compare alternatives. By using *Ω*, a decision maker provides a DPR *d*_12_ related to two alternatives *x*_1_ and *x*_2_, which is *d*_12_ = {(*H*_4_,0.4),(*H*_6_,0.1),(*H*_7_,0.2), (*H*_8_,0.1),(*Ω*,0.2)}. This means that the decision maker is 40% sure *x*_1_ is moderately inferior than *x*_2_, 10% sure *x*_1_ is indifferent to *x*_2_, 30% sure *x*_1_ is marginally superior than *x*_2_, 10% sure *x*_1_ is moderately superior than *x*_2_, and 20% unsure about the relation between *x*_1_ and *x*_2_.

From the DPR *d*_12_ in Example 1, it may be difficult to directly determine whether *x*_1_ is preferred to *x*_2_. To facilitate the comparison between alternatives, Fu et al. (2016) [32] proposed to transform a DPR matrix into a score matrix by using the score values of grades.

**Definition 2.** [32] Let *Ω* = {*H*_1_,*H*_2_,…,*H*_*N*_} be a set of symmetrical grades, as presented in Definition 1. The score value of *H*_*n*_ represented by *s*(*H*_*n*_) is given to profile the characteristics of *H*_*n*_, satisfying

$$0<s(H_{(N+1)/2+1})\;<\;\ldots \;<s(H_{N})=\;1,$$ (6)

$$s(H_{(N+1)/2})=0,$$ (7)

and

$$s(H_{n})=-s(H_{N}{}_{-n+1})\;,\text{for}n\in \{1,\;\ldots ,N\}.$$ (8)

The score value of grade *H*_*n*_, *s*(*H*_*n*_), presented in Definition 2 represents the preference intensity of the grade. It is practically specified by decision makers to reflect their preferences. Some methods of eliciting utilities, such as the probability assignment approach [34], can be used to help determine *s*(*H*_*n*_).

With the aid of *s*(*H*_*n*_), a DPR matrix is transformed into a score matrix *S* ⊂ *X* × *X* with *S* = (*s*_*ij*_)_*M*×*M*_. Without loss of generality, we assume that *m*_*ij*_(*Ω*) > 0. Under this assumption, we have $s_{ij}=\;[s_{ij}^{-},\;\;s_{ij}^{+}]$ with $s_{ij}^{-}=\sum_{n=1}^{N}m_{ij}(H_{n})\cdot s(H_{n})+m_{ij}(\Omega )\cdot s\left(H_{1}\right)$ and $s_{ij}^{+}=\sum_{n=1}^{N}m_{ij}(H_{n})\cdot s(H_{n})+m_{ij}(\Omega )\cdot s(H_{N})$. The calculation follows the principle that *m*_*ij*_(*Ω*) could be assigned to any grade. Reconsidering the DPR *d*_12_ in Example 1, we can obtain that $s_{12}^{-}=0.2\cdot s(H_{7})+0.1\cdot s(H_{8})-0.2\cdot s(H_{1})$ and $s_{12}^{+}=0.2\cdot s(H_{7})+0.1\cdot s(H_{8})+0.2\cdot s(H_{1})$.

### Consistency of the score matrix

Consistency is an important requirement that comparisons among alternatives must satisfy. If it is not satisfied, unacceptable or even counterintuitive decision results will be generated from the comparisons. To guarantee the rational decision result derived from DPRs, Fu et al. (2016) [32] designed the additive consistency of score matrix with the consideration of the preferences of decision makers. The additive consistency and its basic are recalled as follows.

**Definition 3.** [34] Given a set of alternatives *X* = {*x*_1_,*x*_2_,…,*x*_*M*_}, suppose that *P* = (*p*_*ij*_)_*M*×*M*_ is a FPR matrix on *X*. Then, *P* is consistent if *p*_*ij*_ + *p*_*jk*_ + *p*_*ki*_ = 1.5, for *i*,*j*,*k* ∈ {1,…,*M*}.

The consistency condition between FPRs presented in Definition 3 is strict. It is under the assumption that the decision maker providing the FPRs is perfectly rational. In practice, a decision maker is usually of bounded rationality and difficult to provide perfectly consistent FPRs. Meanwhile, the consistency condition presented in Definition 3 may result in the FPR that is not limited to [0,1]. For example, given a set of alternatives *X* = {*x*_1_,*x*_2_,*x*_3_}, we can calculate by using Definition 3 that *p*_13_ = −0.1 from *p*_12_ = 0.8 and *p*_23_ = 0.8. Although the three FPRs are consistent according to Definition 3, *p*_13_ is not limited to [0,1]. To reflect the bounded rationality of a decision maker and avoid generating the unexpected result from the consistency condition, Fu et al. (2016) [32] developed the additive consistency of score matrix.

**Definition 4.** [32] Given a set of alternatives *X* = {*x*_1_,*x*_2_,…,*x*_*M*_}, suppose that $S={\left(s_{ij}=[s_{ij}^{-},\;s_{ij}^{+}]\right)}_{M}{}_{\times M}$ denotes the score matrix obtained from a DPR matrix on *X*. Then, by using a function *f*:[−1,1] × [−1,1] → [−1,1], *S* is defined to be consistent if

$$f(s_{ij}^{-},s_{jk}^{-})+s_{ki}^{+}=0,\;\text{for}\;i,j,k\in \{1,\ldots ,M\},$$ (9)

and

$$f(s_{ij}^{+},s_{jk}^{+})+s_{ki}^{-}=0,\;\text{for}\;i,j,k\in \{1,\ldots ,M\}.$$ (10)

The relevant properties of the function *f* are presented by Fu et al. (2016) [32]. In practical applications, many specific functions satisfying the properties can be used to implement the additive consistency of score matrix. A specific function used by Fu et al. (2016) [32] is given as

$$f_{N}(\text{x},\text{y})=\{\begin{array}{ll} \frac{x+y-(1+a)\cdot xy}{1-a\cdot xy}, & \text{if}\;x\in [0,\;\;1]\;\;\text{and}\;y\in [0,\;\;1]\\ \frac{x+y+(1+a)\cdot xy}{1-a\cdot xy}, & \text{if}\;x\in [-1,\;0]\;\text{and}\;y\in [-1,\;0]\\ x\;+\;y, & \text{otherwise} \end{array}$$ (11)

In real applications, such as decision making, *a* can be determined from $s_{ij}^{-}$, $s_{jk}^{-}$, $s_{ki}^{-}$, $s_{ij}^{+}$, $s_{jk}^{+}$, and $s_{ki}^{+}$ provided by a decision maker when $s_{ij}^{-}\cdot s_{\text{jk}}^{-}>0$ and $s_{ij}^{\text{+}}\cdot s_{\text{jk}}^{\text{+}}>0$. After that, with the help of *f*_*N*_(*x*,*y*), the DPRs between non-neighboring alternatives can be generated from those between neighboring alternatives provided by the decision maker.

## IDPR

In this section, IDPR as a generalization of DPR is proposed and its relevant concepts are defined including score values of IDPRs, their comparison, and their consistency.

### Basic concept of IDPR

From Definition 1, it can be inferred that DPR is often used to help a decision maker express the preferred, non-preferred, indifferent, and uncertain degrees of an alternative over another simultaneously. The four types of degrees are characterized by precise numbers in DPR. However, DPR cannot be suitable for all cases in practice. For example, a DPR cannot be used to compare different radiologists’ capabilities of diagnosing thyroid cancers, as presented in Introduction. To handle such cases, the concept of IDPR is developed.

**Definition 5.** Given a set of alternatives *X* = {*x*_1_,*x*_2_,…,*x*_*M*_} and a set of grades *Ω* = {*H*_1_,*H*_2_,…,*H*_*N*_} with an odd number *N*, suppose that the meaning of grade *H*_*n*_ is presented as Definition 1. The comparison between two alternatives *x*_*i*_ and *x*_*j*_ can then be profiled by an IDPR denoted by $\;r_{ij}=\{(H_{n},\;[m_{ij}^{-}(H_{n}),\;m_{ij}^{+}(H_{n})]),n=\;1,\;\ldots ,N;\;(\Omega ,\;[m_{ij}^{-}(\Omega ),\;m_{ij}^{+}(\Omega )])\}$, where $\left[m_{ij}^{-}(H_{n}),\;m_{ij}^{+}(H_{n})\right]$ stands for the interval-valued belief degree assessed to grade *H*_*n*_ and $\left[m_{ij}^{-}(\Omega ),\;m_{ij}^{+}(\Omega )\right]$ for the interval-valued belief degree of global ignorance between *x*_*i*_ and *x*_*j*_. For *r*_*ij*_, it should be satisfied that

$$0\leq m_{ij}^{-}(H_{n})\leq m_{ij}^{+}(H_{n})\leq 1,\;\text{for}\;\;n\;\in \left\{1,\;\ldots ,N\right\},$$ (12)

$$\sum_{n=1}^{N}m_{ij}^{-}(H_{n})\leq 1,$$ (13)

$$\sum_{n=1}^{N}m_{ij}^{+}(H_{n})\geq \sum_{n=1}^{N}m_{ij}^{-}(H_{n})$$ (14)

$$m_{ij}^{-}(\Omega )=\text{max}(0,1-\sum_{n=1}^{N}m_{ij}^{+}(H_{n})),$$ (15)

$$m_{ij}^{+}(\Omega )=1-\sum_{n=1}^{N}m_{ij}^{-}(H_{n}),$$ (16)

$$[m_{ij}^{-}(H_{n}),m_{ij}^{+}(H_{n})]=[m_{ji}^{-}(H_{N-n+1}),m_{ji}^{+}(H_{N-n+1})],\;\text{for}\;n\in \;\left\{1,\;\ldots ,N\right\},$$ (17)

$$[m_{ij}^{-}(\Omega ),m_{ij}^{+}(\Omega )]=[m_{ji}^{-}(\Omega ),m_{ji}^{+}(\Omega )],$$ (18)

and

$$m_{ii}^{-}(H_{(N+1)/2})=m_{ii}^{+}(H_{(N+1)/2})=1.$$ (19)

When any two alternatives are compared by using IDPRs, an IDPR matrix *R* = (*r*_*ij*_)_*M*×*M*_ ⊂ *X* × *X* is formed. Any element in the IDPR matrix satisfies some properties.

**Remark 1.** For an IDPR, if $m_{ij}^{-}({\text{H}}_{n})=m_{ij}^{+}({\text{H}}_{n})$ and $m_{ij}^{-}(\Omega )=m_{ij}^{+}(\Omega )$, for *n* = 1,…,*N*, then the IDPR reduces to a DPR.

**Remark 2.** For an IDPR, if $\sum_{n=1}^{\text{N}}m_{ij}^{-}({\text{H}}_{n})+m_{ij}^{-}(\Omega )>1$ or $\sum_{n=1}^{\text{N}}m_{ij}^{+}({\text{H}}_{n})+m_{ij}^{+}(\Omega )<1$, then the IDPR is said to be invalid; otherwise, it is valid.

**Remark 3.** For an IDPR, if $\left[m_{ij}^{-}(\Omega ),m_{ij}^{+}(\Omega )]\neq [0,0\right]$, then the IDPR is said to be incomplete; otherwise, it is said to be complete.

**Remark 4.**Given a valid IDPR $\left\{(H_{n},\;[m_{ij}^{-}(H_{n}),m_{ij}^{+}(H_{n})]),n=\;1,\;\ldots ,N;(\Omega ,\;[m_{ij}^{-}(\Omega ),m_{ij}^{+}(\Omega )])\right\}$, any DPR limited to the IDPR denoted by {(*H*_*n*_,*m*_*ij*_(*H*_*n*_),*n* = 1,…,*N*;(*Ω*, *m*_*ij*_(*Ω*)} satisfies that $m_{ij}(H_{n})\;\in \;[m_{ij}^{-}(H_{n}),\;m_{ij}^{+}(H_{n})]\;(\text{n}=1,\ldots ,\text{N}),\;m_{ij}(\Omega )\in [m_{ij}^{-}(\Omega ),\;m_{ij}^{+}(\Omega )]$, and $\begin{array}{ll} \sum_{n=1}^{\text{N}}m_{ij}({\text{H}}_{n})\;\;+\; & m_{ij}(\Omega )=1 \end{array}$.

As indicated by Wang et al. (2006) [35], a normalized interval-valued belief distribution is a valid one, but the converse is not always the case. There is a similar conclusion on IDPRs.

**Definition 6.** Given a valid IDPR $\left\{(H_{n},\;[m_{ij}^{-}(H_{n}),\;m_{ij}^{+}(H_{n})]),n=\;1,\;\ldots ,N;\;(\Omega ,\;[m_{ij}^{-}(\Omega ),\;m_{ij}^{+}(\Omega )])\right\}$, it is said to be a non-normalized one when

$$\sum_{n=1}^{\text{N}}m_{ij}^{-}({\text{H}}_{n})+m_{ij}^{-}(\Omega )+\;(m_{ij}^{+}(H_{m})-m_{ij}^{-}(H_{m}))\geq 1,\;\text{for}\;m\;\in \left\{1,\;\ldots ,N\right\},$$ (20)

and

$$\sum_{n=1}^{\text{N}}m_{ij}^{+}({\text{H}}_{n})+m_{ij}^{+}(\Omega )-(m_{ij}^{+}(H_{m})-m_{ij}^{-}(H_{m}))\leq 1.$$ (21)

A normalized IDPR is a valid one, but a valid IDPR may be a non-normalized one. This is explained by using the following example.

**Example 2.** Suppose that *Ω* = {*H*_1_,*H*_2_,…,*H*_11_} = {absolutely inferior, much more inferior, more inferior, moderately inferior, marginally inferior, indifferent, marginally superior, moderately superior, more superior, much more superior, absolutely superior} denotes a set of grades used to compare alternatives. By using *Ω*, a decision maker provides two IDPRs *r*_12_ and *r*_23_ related to three alternatives *x*_1_, *x*_2_, and *x*_3_, which are

$$r_{12}=\{(H_{4},[0.4,\;0.6]),\;(H_{5},[0.4,\;0.5]),\;(\Omega ,[0.1,\;0.5])\},$$

and

$$r_{23}=\{(H_{5},[0.3,\;0.4]),\;(H_{6},[0.3,\;0.5]),\;(\Omega ,[0.1,\;0.4])\}.$$

From Remark 2, it can be known that both *r*_12_ and *r*_23_ are valid. On the one hand, for *r*_12_, we have $m_{12}^{-}(H_{4})+m_{12}^{-}(H_{5})+m_{12}^{-}(\Omega )+(m_{12}^{+}(H_{4})-m_{12}^{-}(H_{4}))=1.1>1$. According to Definition 6, *r*_12_ is non-normalized. On the other hand, it can be inferred that *r*_23_ is normalized from the following equations.

$$m_{23}^{-}(H_{5})+m_{23}^{-}(H_{6})+m_{23}^{-}(\Omega )+(m_{23}^{+}(H_{5})-m_{23}^{-}(H_{5})))=0.8<1,$$

$$m_{23}^{-}(H_{5})+m_{23}^{-}(H_{6})+m_{23}^{-}(\Omega )+(m_{23}^{+}(H_{6})-m_{23}^{-}(H_{6})))=1\leq 1,$$

$$m_{23}^{+}(H_{5})+m_{23}^{+}(H_{6})+m_{23}^{+}(\Omega )+(m_{23}^{+}(H_{5})-m_{23}^{-}(H_{5}))=1.2>1,$$

and

$$m_{23}^{+}(H_{5})+m_{23}^{+}(H_{6})+m_{23}^{+}(\Omega )+(m_{23}^{+}(H_{6})-m_{23}^{-}(H_{6}))=1.1>1.$$

From what has been analyzed above, for a normalized IDPR $\left\{(H_{n},\;[m_{ij}^{-}(H_{n}),\;m_{ij}^{+}(H_{n})]),n=1,\;\ldots ,N;(\Omega ,\;[m_{ij}^{-}(\Omega ),\;m_{ij}^{+}(\Omega )])\right\}$, given any $m_{ij}(H_{n})\in [m_{ij}^{-}(H_{n}),\;m_{ij}^{+}(H_{n})]$, we can certainly find a DPR limited to the IDPR. If the IDPR is non-normalized, it cannot be done in many situations. For this reason, a normalization formula of IDPR is developed to transform non-normalized IDPRs into normalized ones. In addition, the normalization process of IDPR is illustrated by the following example.

**Remark 5.** For a valid and non-normalized IDPR $\left\{(H_{n},\;[m_{ij}^{-}(H_{n}),\;m_{ij}^{+}(H_{n})]),\;n=1,\ldots ,N;(\Omega ,\;[m_{ij}^{-}(\Omega ),\;m_{ij}^{+}(\Omega )])\right\}$, it can be transformed into a normalized one $\left\{(H_{n},[\text{max}(m_{ij}^{-}(H_{n}),1-(\sum_{m\neq n}m_{ij}^{+}(H_{m})+m_{ij}^{+}(\Omega ))),\text{min}(m_{ij}^{+}(H_{n}),1-(\sum_{m\neq n}m_{ij}^{-}(H_{m})+m_{ij}^{-}(\Omega )))]),n=1,\;\ldots ,N;(\Omega ,\;[m_{ij}^{-}(\Omega ),\;m_{ij}^{+}(\Omega )])\right\}$ The following example is given to explain Remark 5.

**Example 3.** Given a valid and non-normalized IDPR *r*_12_ = {(*H*_4_,[0.4, 0.6]),(*H*_5_,[0.4, 0.5]), (*Ω*,[0.1, 0.5])}, by using Remark 5 the IDPR can be transformed into a valid and normalized one {(*H*_4_,[0.4, 0.5]),(*H*_5_,[0.4, 0.5]), (*Ω*,[0, 0.2])}. The detailed transformation process is presented in the following:

$$m_{12}^{-}(H_{4})=\text{max}(m_{12}^{-}(H_{4}),1-(\sum_{m\neq 4}m_{12}^{+}(H_{m})+m_{12}^{+}(\Omega )=\text{max}(0.4,0.3)=0.4,$$

$$m_{12}^{\text{+}}(H_{4})=\text{min}(m_{12}^{\text{+}}(H_{4}),1-(\sum_{m\neq 4}m_{12}^{-}(H_{m})+m_{12}^{-}(\Omega ))=\text{min}(0.6,0.5)=0.5,$$

$$m_{12}^{-}(H_{5})=\text{max}(m_{12}^{-}(H_{5}),1-(\sum_{m\neq 5}m_{12}^{+}(H_{m})+m_{12}^{+}(\Omega ))=\text{max}(0.4,-0.1)=0.4,$$

$$m_{12}^{\text{+}}(H_{5})=\text{min}(m_{12}^{\text{+}}(H_{5}),1-(\sum_{m\neq 5}m_{12}^{-}(H_{m})+m_{12}^{-}(\Omega ))=\text{min}(0.5,0.5)=0.5,$$

$$m_{12}^{-}(\Omega )=\text{max}(0,\;1-m_{12}^{\text{+}}(H_{4})-m_{12}^{\text{+}}(H_{5}))=\text{max}(0,0)=0,$$

and

$$m_{12}^{+}(\Omega )=1-m_{12}^{-}(H_{4})-m_{12}^{-}(H_{5})=0.2.$$

### Comparison of the score intervals of IDPRs

To facilitate the comparison between alternatives, a DPR matrix is transformed into its score matrix with the aid of score values of grades, as presented in Definition 2. For the same purpose, an IDPR matrix can be transformed into a score matrix. As the belief degree assigned to *H*_*n*_(*n* = 1,…,*N*) or *Ω* in an IDPR is an interval instead of a precise number, the following optimization models are constructed to transform the IDPR to its score interval denoted by $\left[s_{ij}^{-},\;s_{ij}^{+}\right]$.

$$\text{MIN}\;\;\;s_{ij}=\sum_{n=1}^{N}m_{ij}^{}(H_{n})\cdot s(H_{n})+m_{ij}(\Omega )\cdot s(H_{1})$$ (22)

$$\text{s}.\text{t}.\;\;\;\;\;m_{ij}^{-}(H_{n})\leq m_{ij}(H_{n})\leq m_{ij}^{+}(H_{n}),n=1,\ldots ,N,$$ (23)

$$m_{ij}^{-}(\Omega )\;\leq m_{ij}(\Omega )\;\leq m_{ij}^{+}(\Omega ),$$ (24)

and

$$\sum_{n=1}^{N}m_{ij}^{}(H_{n})+m_{ij}(\Omega )\;=\;1.$$ (25)

$$\text{MAX}\;\;\;s_{ij}=\sum_{n=1}^{N}m_{ij}^{}(H_{n})\cdot s(H_{n})+m_{ij}(\Omega )\cdot s(H_{N})$$ (26)

$$\text{s}.\text{t}.\;\;\;\;\;m_{ij}^{-}(H_{n})\leq m_{ij}(H_{n})\leq m_{ij}^{+}(H_{n}),n=1,\ldots ,N,$$ (27)

$$m_{ij}^{-}(\Omega )\;\leq m_{ij}(\Omega )\;\leq m_{ij}^{+}(\Omega ),$$ (28)

and

$$\sum_{n=1}^{N}m_{ij}^{}(H_{n})+m_{ij}(\Omega )=1.$$ (29)

Solving the optimization models shown in Eqs (22)–(25) and (26)–(29) generates $s_{ij}^{-}$ and $s_{ij}^{+}$. When all elements in an IDPR matrix are transformed into their score intervals, the IDPR matrix is transformed into its score matrix denoted by $S={\left(s_{ij}=[s_{ij}^{-},\;s_{ij}^{+}]\right)}_{M\times M}\subset X\times X$. From Definitions 2 and 5 and the above two optimization models, it can be known that $\left[s_{ij}^{-},\;s_{ij}^{+}]\subseteq [-1,\;1\right]$. Not only that, $\left[s_{ij}^{-},\;s_{ij}^{+}\right]$ has the following properties.

**Proposition 1.** Suppose that *X* = {*x*_1_,*x*_2_,…,*x*_*M*_} is a set of alternatives and $S={\left(s_{ij}=[s_{ij}^{-},\;s_{ij}^{+}]\right)}_{M\times M}$ is the score matrix derived from the IDPR matrix on *X*. Then *S* satisfies

$$s_{ij}^{-}+s_{ji}^{+}=0,\text{for}\;i,j\in \{1,\;\ldots ,M\},$$ (30)

and

$$s_{ij}^{+}+s_{ji}^{-}=0,\text{for}\;i,j\in \{1,\;\ldots ,M\}.$$ (31)

Proof of Proposition 1 is presented in Appendix A in S1 File. When any element in an IDPR matrix is transformed into its score interval, we can compare score intervals $\left[s_{ij}^{-},\;s_{ij}^{+}\right]$ and $\left[s_{ij}^{-},\;s_{ij}^{+}\right]$ to determine the relationship between alternatives *x*_*i*_ and *x*_*j*_ with the aid of the method of comparing two interval numbers developed by Zhang et al (1999) [36].

**Definition 7.** When score intervals $s_{ij}=[s_{ij}^{-},\;s_{ij}^{+}]$ and $s_{ji}=[s_{ji}^{-},\;s_{ji}^{+}]$ are obtained, the possibility degree of *s*_*ij*_ ≥ *s*_*ji*_ is defined as

$$p(s_{ij}\geq s_{ji})=\{\begin{array}{ll} 1, & \;\;\;\;\;\;\;s_{ij}^{-}>s_{ji}^{+}\\ 1-\frac{2{\left(s_{ij}^{-}\right)}^{2}}{{\left(s_{ij}^{+}-s_{ij}^{-}\right)}^{2}}, & s_{ji}^{-}\leq s_{ij}^{-}\leq s_{ji}^{+}\leq s_{ij}^{+}\\ \frac{2{\left(s_{ij}^{-}\right)}^{2}}{{\left(s_{ij}^{+}-s_{ij}^{-}\right)}^{2}},\; & s_{ij}^{-}\leq s_{ji}^{-}\leq s_{ij}^{+}\leq s_{ji}^{+}\\ 0, & \;\;\;\;\;\;\;s_{ij}^{+}<s_{ji}^{-} \end{array}$$ (32)

When the possibility degree between any two alternatives is calculated by using Definition 7, the possibility degree matrix *P* = (*p*(*s*_*ij*_ ≥ *s*_*ji*_))_*M*×*M*_ is obtained. From the possibility matrix, the relationship between any two alternatives can be determined.

**Definition 8.** Given a set of alternatives *X* = {*x*_1_,*x*_2_,…,*x*_*M*_}, suppose that the score matrix *S* is obtained from an IDPR matrix on *X*. Then the relationship between alternatives *x*_*i*_ and *x*_*j*_ can be determined by the possibility degree *p*(*s*_*ij*_ ≥ *s*_*ji*_), which is calculated by using Definition 8.

1. *x*_*i*_ is absolutely superior to *x*_*j*_ if *p*(*s*_*ij*_ ≥ *s*_*ji*_) = 1 and $s_{ij}^{-}>0$, which is denoted by $x_{i}\overset{1}{≻}x_{j}$.
2. *x*_*i*_ is superior to *x*_*j*_ with the possibility degree *p*(*s*_*ij*_ ≥ *s*_*ji*_) if 0.5 < *p*(*s*_*ij*_ ≥ *s*_*ji*_) < 1 or 0 < *p*(*s*_*ij*_ ≥ *s*_*ji*_) < 0.5, which is denoted by $x_{i}\overset{p(s_{ij}\geq s_{ji})}{≻}x_{j}$.
3. *x*_*i*_ is equal to *x*_*j*_ if the possibility degree *p*(*s*_*ij*_ ≥ *s*_*ji*_) = 0.5 and $s_{ij}^{-}+s_{ij}^{+}=0$, which is denoted by $x_{i}\overset{0.5}{\approx }x_{j}$.
4. *x*_*i*_ is absolutely inferior to *x*_*j*_ if *p*(*s*_*ij*_ ≥ *s*_*ji*_) = 0 and $s_{ij}^{\text{+}}<0$, which is denoted by $x_{i}\overset{0}{≻}x_{j}$.

### Consistency of the score matrix of IDPRs

When the score matrix is obtained from an IDPR matrix, it can be used to compare any two alternatives, as presented in Definition 8. The rationality of all comparisons between alternatives is closely associated with the consistency of the score matrix. Inconsistent score matrix may result in the conflict in the comparisons between alternatives. To guarantee rational comparisons between alternatives, in the following we discuss the consistency of score intervals among any three alternatives.

The concept of consistency is closely associated with transitivity [33]. Different types of transitivity, such as max transitivity, min transitivity, max-min transitivity, additive transitivity, and multiplicative transitivity [34, 37–39], result in different types of consistency. In theory, any type of consistency can be used to guarantee rational comparisons between alternatives. As a representative consistency developed from additive transitivity, additive consistency has been widely applied in modeling the consistency of different types of preference relations. More importantly, the additive consistency of a DPR matrix is constructed by Fu et al. (2016) [32], as presented in Definition 4. To be consistent with the situation of DPR, the additive consistency of an IDPR matrix is defined.

**Definition 9.** Given a set of alternatives *X* = {*x*_1_,*x*_2_,…,*x*_*M*_}, the score matrix $S={\left(s_{ij}=[s_{ij}^{-},s_{ij}^{+}]\right)}_{M\times M}$ derived from an IDPR matrix on *X* is defined to be additive consistent if

$$f(s_{ij}^{-},s_{jk}^{-})+s_{ki}^{+}=0,\;\text{for}\;i,j,k\in \{1,\ldots ,M\},$$ (33)

and

$$f(s_{ij}^{+},s_{jk}^{+})+s_{ki}^{-}=0,\text{for}\;i,j,k\in \{1,\ldots ,M\}.$$ (34)

where *f*:[−1,1]×[−1,1]→[−1,1] is a binary function.

The function *f* used to construct the additive consistency satisfies the following properties.

**Property 1.** [33,40–44] Given *x* and *y* such that *x* ∈ [−1,1] and *y* ∈ [−1,1], a function *f*:[−1,1]×[−1,1]→[−1,1] used to combine *x* with *y* satisfies

1. (Max transitivity) *f*(*x*,*y*) ≥ max{*x*,*y*}, if *x* ∈ (0,1) and *y* ∈ (0,1),
2. (Min transitivity) *f*(*x*,*y*) ≤ min{*x*,*y*}, if *x* ∈ (−1,0) and *y* ∈ (−1,0),
3. (Max-min transitivity) min{*x*,*y*} ≤ *f*(*x*,*y*) ≤ max{*x*,*y*} if *x* · *y* < 0,
4. (Monotonicity) $f(x,y)\leq f(\bar{x},y),\;\text{if}x\leq \bar{x},x\in (-1,\;1),\bar{x}\in (-1,\;1)\text{and}y\in (-1,\;1),$
5. (Monotonicity) $f(x,y)\leq f(x,\bar{y}),\;\text{if}y\leq \bar{y},x\in (-1,\;1),y\in (-1,\;1),\;\text{and},\bar{y}\in (-1,\;1),$
6. (Continuity) $\underset{\begin{array}{l} x\to x^{\prime }\\ y\to y^{\prime } \end{array}}{\text{lim}}f(x,\;y)=f(x^{\prime },y^{\prime }),\;\text{for}x\in [-1,\;1]\;\text{and}y\in [-1,\;1],$
7. (Extreme element) *f*(1,*x*) = *f*(*y*,1) = 1 if *x* ∈ (0,1] and *y* ∈ (0,1],
8. (Extreme element) *f*(−1,*x*) = *f*(*y*,−1) = −1, if *x* ∈ [−1,0) and *y* ∈ [−1,0), and
9. (Neutral element) *f*(*x*,0) = *x*, if *x* ∈ [−1,1]; and *f*(0,*y*) = *y*, if *y* ∈ [−1,1].

To develop the additive consistency of the score matrix transformed from an IDPR matrix, the function *f* satisfying Property 1 is defined as follows.

**Definition 10.** Suppose that *f* is the function used to design the additive consistency of the score matrix derived from an IDPR matrix on a set of alternatives *X* = {*x*_1_,*x*_2_,…,*x*_*M*_}, then the function *f* is defined as

f(x,y)=p(x,y),if0≤x⋅y≤1,x≥0andy≥0, (35)

f(x,y)=−p(−x,−y),if0≤x⋅y≤1,x≤0andy≥0, (36)

and

f(x,y)=q(x,y)if−1≤x⋅y<0. (37)

In Definition 10, functions *p* and *q* used to construct *f*(*x*,*y*) can be $p(x,y)=\frac{x+y-(1+a)\cdot xy}{1-a\cdot xy}$ and $q(x,y)=\frac{\left(1-b)\cdot (x+y\right)}{1-b\cdot xy}$, where *a* ∈ (−∞, 1) and *b* ∈ [0,1] are the parameters. The difference between two functions in Definition 4 and Definition 10 is that the function *q* replaces the function *x* + *y* in the case of −1 ≤ *x* · *y* < 0. In what follows, we focus on the function *q* and its parameter *b* to analyze their basic properties.

**Proposition 2.** Suppose that $q(x,y)=\frac{\left(1-b)\cdot (x+y\right)}{1-b\cdot xy}$ is a two-variable function with a parameter *b* ∈ [0,1]. Then, the function *q* satisfies

min{x,y}≤q(x,y)≤max{x,y},if−1≤x⋅y<0, (38)

$$q(x,y_{1})>q(x,y_{2}),\;\text{if}\;-1\leq x\cdot y<0\;\text{and}y_{2}<y_{1},$$ (39)

and

$$q(x_{1},y)>q(x_{2},y),\;if-1\leq x\cdot y<0\;\text{and}x_{2}<x_{1}.$$ (40)

Proof of Proposition 2 is presented in Appendix A in S1 File. When −1 ≤ *x* · *y* < 0, the influence of the parameter *b* on *q*(*x*,*y*) is analyzed in the following proposition.

**Proposition 3.** Suppose that $q(x,y)=\frac{\left(1-b)\cdot (x+y\right)}{1-b\cdot xy}$ is a two-variable function with a parameter *b* ∈ [0,1]. Then, if *x* + *y* ≥ 0, the function is monotonously decreasing with respect to *b*. Otherwise, the function is monotonously increasing with respect to *b*.

Proof of Proposition 3 is presented in Appendix A in S1 File. In real applications, the parameters *a* and *b* can be determined in accordance with the preferences of decision makers, which will be demonstrated in Eqs (41)–(53). In what follows, the relationship between the additive consistency in Definition 10 and the additive consistency in Definition 3 is discussed. Before discussing the relationship, a function *t*(*x*) = 2(*x* − 0.5) with *x* ∈ [0.1] is used to unify the value ranges of FPR and the function *f*.

**Theorem 1.** Suppose that *x*, *y*, and *z* are three variables such that *x* ∈ [0.1], *y* ∈ [0.1], *z* ∈ [0.1], and *t*(*x*) · *t*(*y*) <0, then *x* + *y* + *z* = 1.5 is a special case of *f*(*t*(*x*), *t*(*y*)) + *t*(*z*) = 0.

Proof of Theorems 1 is presented in Appendix A in S1 File. Theorems 1 reveals that if *t*(*x*) · *t*(*y*) <0, i.e., *x* < 0.5 and *y* > 0.5 or *x* > 0.5 and *y* < 0.5, *x* + *y* + *z* = 1.5 may be greater than, equal to, or less than *f*(*t*(*x*), *t*(*y*)) + *t*(*z*) = 0 when *b* takes different values within the interval [0,1]. On the contrary, if *t*(*x*) · *t*(*y*) ≥ 0, i.e., *x* ≤ 0.5 and *y* ≤ 0.5 or *x* ≥ 0.5 and *y* ≥ 0.5, *x* + *y* + *z* = 1.5 can also be seen as a special case of *f*(*t*(*x*), *t*(*y*)) + *t*(*z*) = 0, which was discussed by Fu et al. (2016) [32] and thus is omitted here. From what has been analyzed above, we can know that the parameters *a* and *b* of the function *f* play an important role in the modeling of the additive consistency of the score matrix transformed from an IDPR matrix.

The contents in this section indicate that IDPRs can be rationally applied in practice under the assumption that the provided IDPRs among alternatives satisfy the additive consistency that is presented in Definition 9. In what follows, the determination of the parameters *a* and *b* in MCGDM will be investigated.

## Application of IDPRs to MCGDM

With the consideration of the concept of IDPR, the comparison of score intervals of IDPRs, and the consistency among the score intervals of IDPRs, IDPRs are applied to model and analyze MCGDM problems in this section.

### Modeling of a MCGDM problem by using IDPRs

Suppose that a MCGDM problem includes a facilitator, *T* decision makers *t*_*j*_(*j* = 1,…*T*), *M* alternatives *a*_*l*_(*l* = 1,…*M*), and *L* criteria *e*_*i*_(*i* = 1,…*L*). The relative weights of *T* decision makers on criterion *e*_*i*_ are denoted as *λ*(*e*_*i*_) = {*λ*^1^(*e*_*i*_),…,*λ*^*j*^(*e*_*i*_),…,*λ*^*T*^(*e*_*i*_)}, satisfying 0 ≤ *λ*^*j*^(*e*_*i*_) ≤ 1 and $\sum_{j=1}^{T}\lambda^{j}(e_{i})=1$. The relative weights of the *L* criteria provided by the *T* decision makers are denoted as *w* = (*w*_1_,…,*w*_*i*_,…,*w*_*L*_), satisfying 0 ≤ *w*_*i*_ ≤ 1 and $\sum_{i=1}^{L}w_{i}=1$. Through considering the different background, knowledge, and experience of the four decision makers, the facilitator can specify the relative weights of decision makers on each criterion with the aid of some methods, such as the method developed by Ölçer and Odabaşi (2005) [45]. In a similar way, the relative weights of criteria can be assigned by the facilitator. If all decision makers participate in the determination of criterion weights, they must reach a consensus on the weights.

Given a set of grades *Ω* = {*H*_1_,*H*_2_,…,*H*_*N*_}, decision maker *t*_*j*_ compares alternatives *a*_*l*_ and *a*_*m*_ on criterion *e*_*i*_ to provide the IDPR $r^{j}(e_{i}(a_{l}{}_{m}))=\{(H_{n},\;[\beta_{n,i}^{j-}(a_{lm}),\;\beta_{n,i}^{j+}(a_{lm})]),n=\;1,\;\ldots ,N;\;(\Omega ,\;[\beta_{\Omega ,i}^{j-}(a_{lm}),\;\beta_{\Omega ,i}^{j+}(a_{lm})])\}$. When all pairs of alternatives are compared by *t*_*j*_, an IDPR matrix *R*^*j*^ = (*r*^*j*^(*e*_*i*_(*a*_*lm*_)))_*M*×*M*_ is formed. To facilitate collecting the preferences of decision makers and unifying the cognition of decision makers, the facilitator specifies the score values of grades *s*(*H*_*n*_) with the consideration of the opinions of decision makers.

### Analysis of a MCGDM problem with IDPRs

Facing a MCGDM problem, decision makers need to compare *M* · (*M* − 1) · *L*/2 pairs of alternatives on each criterion. This is a burden on decision makers especially when there are large numbers of alternatives and criteria. More importantly, it is very difficult for decision makers to guarantee the consistency of the comparisons between alternatives. To relieve the burden on decision makers and guarantee the consistency of the score matrix, a feasible way is to require decision makers to provide the comparisons between neighboring alternatives and derive the comparisons between non-neighboring alternatives from the provided ones through consistency, as suggested by Sen and Yang (1994) [46]. By following this principle, (*M* − 1) · *L* IDPRs between neighboring alternatives on each criterion are provided in a MCGDM problem with IDPRs. Note that decision makers provide the comparisons between neighboring alternatives by following an assumption. The assumption is that one decision maker gives the same IDPR between neighboring alternatives on one criterion no matter whether the IDPRs from other decision makers on the criterion and his or her IDPRs on other criteria are known or not. This is to guarantee that the IDPRs from different decision makers and the group IDPRs on different criteria can be combined using the evidential reasoning (ER) algorithm developed by Wang et al. (2006) [35]. After the score intervals of the provided IDPRs are obtained, the score intervals of non-neighboring alternatives can be deduced by using Definition 9.

**Fig 1:**
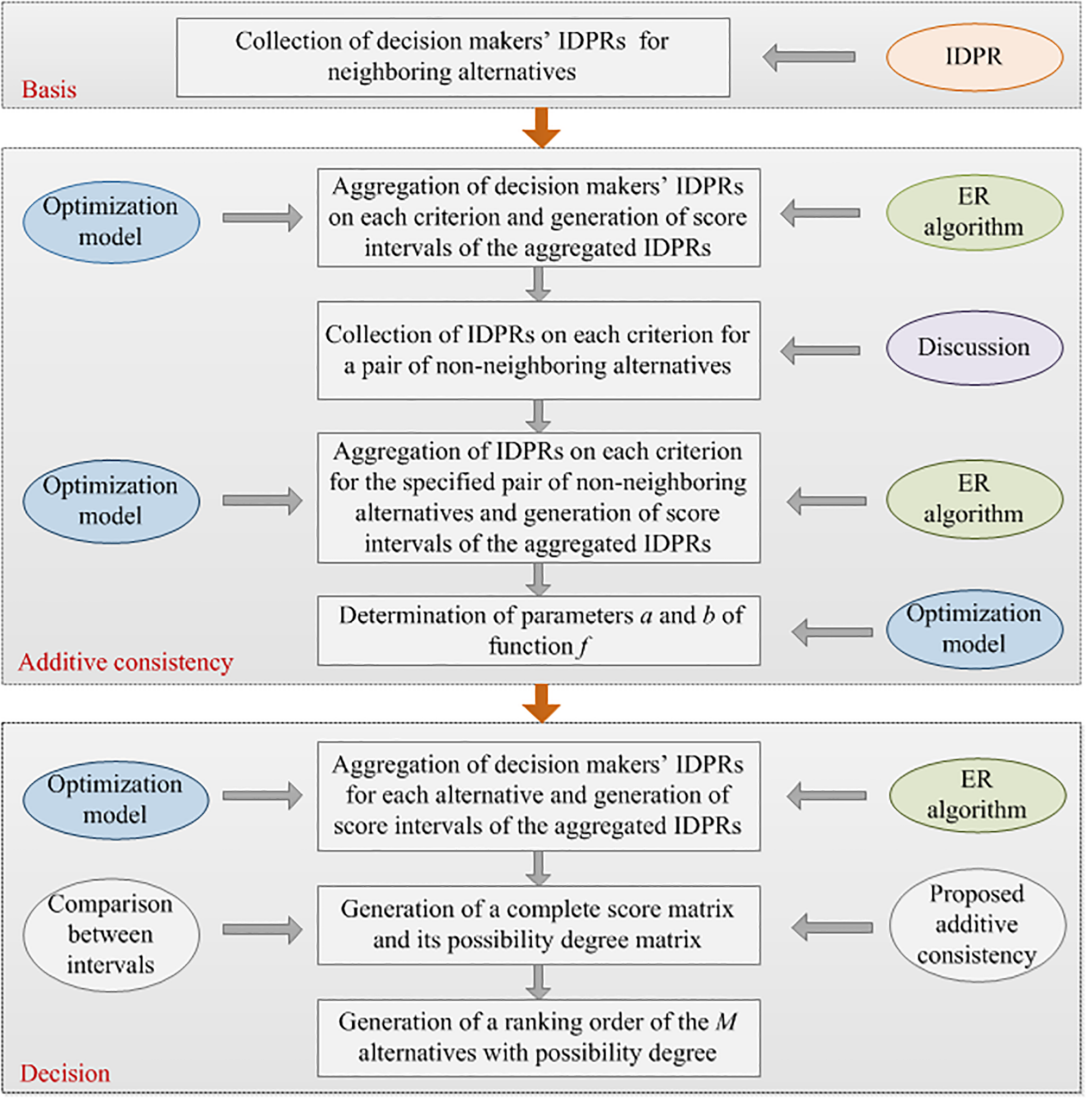
Process of generating a solution to the MCGDM problem with IDPRs.

On the condition that (*M* − 1) · *L* IDPRs of neighboring alternatives on each criterion are provided, the process of generating a solution to the MCGDM problem is described in Fig 1. Regarding this figure as foundations, we analyze the process in the following.

Suppose that the IDPR between neighboring alternatives on criterion *e*_*i*_ provided by decision maker *t*_*j*_ is represented by *r*^*j*^(*e*_*i*_(*a*_*l*(*l*+1)_))(*l* = 1,…,*M*−1). Through the ER algorithm and *λ*(*e*_*i*_), *r*^*j*^(*e*_*i*_(*a*_*l*(*l*+1)_)) is combined to generate the group IDPR *r*(*e*_*i*_(*a*_*l*(*l*+1)_)). Through solving the optimization models shown in Eqs (22)–(25) and (26)–(29) with the help of the score value of *H*_*n*_, the score intervals of the aggregated IDPRs are obtained as *s*(*e*_*i*_(*a*_*l*(*l*+1)_)) = [*s*^−^(*e*_*i*_(*a*_*l*(*l*+1)_)), *s*^+^(*e*_*i*_(*a*_*l*(*l*+1)_))] such that *s*(*e*_*i*_(*a*_*l*(*l*+1)_)) ⊆ [−1,1].

As presented in Definition 10, before the score intervals of IDPRs between non-neighboring alternatives are derived from those between neighboring alternatives, the parameters *a* and *b* of the functions *p* and *q* should be determined. For this purpose, considering the common opinions of decision makers, the facilitator selects two pairs of neighboring alternatives $a_{l^{*}}$ and $a_{l^{*}+1}$ as well as $a_{l^{*}+1}$ and $a_{l^{*}+2}$, and requires decision makers to provide the IDPR between $a_{l^{*}}$ and $a_{l^{*}+2}$, i.e., $r^{j}(e_{i}(a_{l^{*}(l^{*}+2)})$. Similar to the process of *r*^*j*^(*e*_*i*_(*a*_*l*(*l*+1)_)), $r^{j}(e_{i}(a_{l^{*}(l^{*}+2)})$ is combined by using the ER algorithm and *λ*(*e*_*i*_) to generate $r(e_{i}(a_{l^{*}(l^{*}+2)})$. After $r(e_{i}(a_{l^{*}(l^{*}+2)})$ is transformed into its score interval $s(e_{i}(a_{l^{*}(l^{*}+2)})=[s^{-}(e_{i}(a_{l^{*}(l^{*}+2)}),\;s^{\text{+}}(e_{i}(a_{l^{*}(l^{*}+2)})]$, $s(e_{i}(a_{l^{*}(l^{*}+1)})$, $s(e_{i}(a_{\left(l^{*}+1)(l^{*}+2\right)})$, and $s(e_{i}(a_{l^{*}(l^{*}+2)})$ are used to determine the parameters *a* and *b* of the functions *p* and *q* through Definitions 9 and 10.

To simply present the process of determining the parameters *a* and *b*, $s(e_{i}(a_{l^{*}(l^{*}+1)})$, $s(e_{i}(a_{\left(l^{*}+1)(l^{*}+2\right)})$, and $s(e_{i}(a_{l^{*}(l^{*}+2)})(i=1,\ldots ,L)$ are denoted by $s_{i,\;12}^{}=[s_{i,\;12}^{-},\;s_{i,\;12}^{+}]$, $s_{i,\;23}^{}=[s_{i,\;23}^{-},\;s_{i,\;23}^{+}]$, and $s_{i,\;13}^{}=[s_{i,\;13}^{-},\;s_{i,\;13}^{+}]$, respectively. From Definition 10, we can know that the function *p* is employed if $s_{i,\;12}^{-}\cdot s_{i,\;23}^{-}>0$ and $s_{i,\;12}^{+}\cdot s_{i,\;23}^{+}>0$ and the function *q* is employed if $s_{i,\;12}^{-}\cdot s_{i,\;23}^{-}\leq 0$ and $s_{i,\;12}^{+}\cdot s_{i,\;23}^{+}\leq 0$. In the following, an optimization model is constructed to determine the parameters *a* and *b*.

$$\text{MIN}\;\;\sum_{i=1,\;S_{i,\;12}^{-}\cdot S_{i,\;23}^{-}\;>0}^{L}{\left(f^{*}(s_{i,\;13}^{-})-f(|s_{i,\;12}^{-}|,|s_{i,\;23}^{-}|)\right)}^{2}+$$ (41)

$$\sum_{i=1,\;S_{i,\;12}^{+}\cdot S_{i,\;23}^{+}>0}^{L}{\left(f^{*}(s_{i,\;13}^{+})-f(|s_{i,\;12}^{+}|,|s_{i,\;23}^{+}|)\right)}^{2}+$$ (42)

$$\sum_{i=1,\;S_{i,\;12}^{-}\cdot S_{i,\;23}^{-}\;\leq 0}^{L}{\left(f^{*}(s_{i,\;13}^{-})-f(s_{i,\;12}^{-},s_{i,\;23}^{-})\right)}^{2}+$$ (43)

$$\sum_{i=1,\;S_{i,\;12}^{+}\cdot S_{i,\;23}^{+}\leq 0}^{L}{\left(f^{*}(s_{i,\;13}^{+})-f(s_{i,\;12}^{+},s_{i,\;23}^{+})\right)}^{2}$$ (44)

$$\text{s}.\text{t}.\;\;f(\left|s_{i,\;12}^{-}\right|,\left|s_{i,\;23}^{-}\right|)=\frac{\left|s_{i,12\;}^{-}|+|s_{i,23\;}^{-}|-\;(1+a)\cdot |s_{i,12\;}^{-}\cdot s_{i,23\;}^{-}\right|}{1-a\cdot |s_{i,12\;}^{-}\cdot s_{i,23\;}^{-}|}$$ (45)

$$f(\left|s_{i,\;12}^{-}\right|,\left|s_{i,\;23}^{-}\right|)=\frac{\left|s_{i,\;12}^{+}|+|s_{i,\;23}^{+}|-(1+a)\cdot |s_{i,\;12}^{+}\cdot s_{i,\;23}^{+}\right|}{1-a\cdot |s_{i,\;12}^{+}\cdot s_{i,\;23}^{+}|},$$ (46)

$$f(s_{i,\;12}^{-},s_{i,\;23}^{-})=\frac{s_{i,12\;}^{-}+s_{i,23\;}^{-}}{1-b\cdot s_{i,12\;}^{-}\cdot s_{i,23\;}^{-}},$$ (47)

$$f(s_{i,\;12}^{+},s_{i,\;23}^{+})=\frac{s_{i,\;12}^{+}+s_{i,\;23}^{+}}{1-b\cdot s_{i,\;12}^{+}\cdot s_{i,\;23}^{+}},$$ (48)

$$f^{*}(s_{i,13}^{-})=\{\begin{array}{ll} |s_{i,13}^{-}|, & \text{if}\;|s_{i,13}^{-}|\geq \text{max}\{|s_{i,12}^{-}|,|s_{i,23}^{-}|\}>0\\ f(|s_{i,12}^{-}|,|s_{i,23}^{-}|), & \text{otherwise} \end{array},$$ (49)

$$f^{*}(s_{i,\;13}^{+})=\{\begin{array}{ll} |s_{i,\;13}^{+}|, & \text{if}\;|s_{i,\;13}^{+}|\geq \text{max}\{|s_{i,12}^{+}|,|s_{i,\;23}^{+}|\}>0\\ f(|s_{i,12}^{+}|,|s_{i,\;23}^{+}|), & \text{otherwise} \end{array},$$ (50)

$$f^{*}(s_{i,13}^{-})=\{\begin{array}{ll} s_{i,\;13}^{-}, & \text{if}\;\text{min}\;\{s_{i,12}^{-},s_{i,\;23}^{-}\}\leq \;s_{i,\;13}^{-}\leq \text{max}\{s_{i,12}^{-},s_{i,\;23}^{-}\}\\ f(s_{i,12}^{-},s_{i,\;23}^{-}), & \text{otherwise} \end{array},$$ (51)

$$f^{*}(s_{i,\;13}^{+})=\{\begin{array}{ll} s_{i,\;13}^{+}, & \text{if}\;\text{min}\;\{s_{i,12}^{+},s_{i,\;23}^{+}\}\leq \;s_{i,\;13}^{+}\leq \text{max}\{s_{i,12}^{+},s_{i,\;23}^{+}\}\\ f(s_{i,12}^{+},s_{i,\;23}^{+}), & \text{otherwise} \end{array},$$ (52)

−∞<a<1,0≤b≤1. (53)

Through using Definitions 9 and 10 with the aid of the resulting parameters *a* and *b*, *s*(*e*_*i*_(*a*_*lm*_))(*m* ≠ *l* + 1) can be obtained from *s*(*e*_*i*_(*a*_*l*(*l+*1)_)). Unfortunately, *s*(*e*_*i*_(*a*_*lm*_)) cannot be directly used to produce *s*(*a*_*lm*_) and then generate a solution to the MCGDM problem. For this reason, the IDPRs *r*(*e*_*i*_(*a*_*l*(*l+1*)_))(*i* = 1,…,*L*) are combined by using the ER algorithm and *w*_*i*_(*i* = 1,…,*L*) to generate the aggregated IDPR *r*(*a*_*l*(*l+1*)_). The aggregated IDPR is further transformed into its score interval *s*(*a*_*l*(*l+*1)_) through solving the two optimization models shown in Eqs (22)–(25) and (26)–(29).

With the help of the parameters *a* and *b*, the score intervals of non-neighboring alternatives *s*(*a*_*lm*_)(*l* > *m*) are obtained from *s*(*a*_*l*(*l+1*)_) through Definitions 9 and 10. Then, the score intervals of non-neighboring alternatives *s*(*a*_*lm*_)(*l* > *m*) can be obtained from *s*(*a*_*lm*_)(*m* ≥ *l +* 1) in accordance with Proposition 1. From the complete score matrix formed by *s*(*a*_*lm*_)(*m* ≥ *l +* 1) and *s*(*a*_*lm*_)(*l > m*), the possibility degree matrix *P* = (*p*(*s*(*a*_*lm*_) ≥ *s*(*a*_*ml*_)))_*M*×*M*_ (*l*,*m* ∈ {1,…,*M*}) is created by using Definition 7. A ranking order of the *M* alternatives with possibility degrees is then generated from the matrix *P* by using Definition 8, which is considered as a solution to the MCGDM problem.

### Analysis of the parameters associated with the consistency

In the above analysis, the parameters *a* and *b* are obtained through the preferences of the group of decision makers. However, this does not mean that the two parameters cannot be derived from the preferences of each decision maker. When the three IDPRs of decision maker *t*_*j*_, namely *r*^*j*^(*e*_*i*_(*a*_*l*(*l+*1)_)), *r*^*j*^(*e*_*i*_(*a*_(*l+*1)(*l+*2)_)), and *r*^*j*^(*e*_*i*_(*a*_*l*(*l+*2)_)) are provided, their score intervals *s*^*j*^(*e*_*i*_(*a*_*l*(*l+*1)_)), *s*^*j*^(*e*_*i*_(*a*_(*l+*1)(*l+*2)_)), and *s*^*j*^(*e*_*i*_(*a*_*l*(*l+*2)_)) can be obtained and used to determine the two parameters of *t*_*j*_ through solving the optimization model shown in Eqs (41)–(53). An interesting issue is to analyze the relationship between the two parameters associated with each decision maker and those associated with the group.

Suppose that *δ*_*j*_(*δ*_*j*_ ∈ {*a*_*j*_,*b*_*j*_}) represents the two parameters associated with decision maker *t*_*j*_(*j* = 1,…*T*) called individual parameters, and *δ*(*δ* ∈ {*a*,*b*}) represents the two parameters associated with the group called group parameters. As the ER algorithm used to combine the IDPRs of decision makers is a nonlinear one, the relationship between *δ*_*j*_ and *δ* may be nonlinear. In this situation, the least square method is selected to fit the relationship between individual parameters and group parameters. In the following, how to use the least square method to fit the relationship between individual parameters and group parameters is presented.

Although a set of *δ*_*j*_ and *δ* can be used to find the relationship between *δ*_*j*_ and *δ*, the result may be considered the accidental one. To make it more convinced, multiple sets of *δ*_*j*_ and *δ* are encouraged to be used to fit the relationship between individual parameters and group parameters. According to the process of generating a solution to the MCGDM problem by using IDPRs, both *δ*_*j*_ and *δ* are associated with *s*(*H*_*n*_). This provides a feasible way to generate different sets of *δ*_*j*_ and *δ*. Following this way, we use (1−*λ*) · *s*(*H*_*n*_) (−1 ≤ *λ* ≤ 1) to obtain *Γ* sets of *δ*_*j*_ and *δ*. If possible, *Γ* is suggested to be larger than 50. Considering the *Γ* sets of *δ*_*j*_ and *δ* as foundations, we construct the following optimization model to fit the relationship between individual parameters and group parameters.

$$\text{MIN}\;\;\;\theta =\sum_{i=1}^{\Gamma }{\left(g(\;\delta_{1}^{i},\ldots ,\;\delta_{j}^{i},\;\ldots ,\;\delta_{T}^{i})\;-\;\delta^{i}\right)}^{2}$$ (54)

$$\text{s}.\text{t}.\;\;g(\;\delta_{1}^{i},\ldots ,\;\delta_{j}^{i},\;\ldots ,\;\delta_{T}^{i})=\sum_{j=1}^{T}u(\alpha_{j})\cdot v(\delta_{j}^{i}),\;i=1,\;\ldots ,\Gamma ,$$ (55)

$$a_{1}+\ldots +a_{j}+\ldots +a_{T}=1,$$ (56)

$$0\leq a_{j}\leq 1,j=1,\;\ldots ,T,$$ (57)

and

$$\delta_{j}^{i}\in \{a_{j}^{i},b_{j}^{i}\},\;\delta^{i}\in \{a^{i},b^{i}\}.$$ (58)

In the above optimization model, *α*_*j*_(*j* = 1,…,*T*) represents the influence degree of the parameter $\delta_{j}^{i}$ to the parameter *δ*^*i*^, which satisfies that $\sum_{j=1}^{T}\alpha_{j}=1$. Meanwhile, *u*(*a*_*j*_) and $v(\delta_{j}^{i})$ represent the functions of *α*_*j*_ and $\delta_{j}^{i}$, respectively.

## Illustrative example

In this section, the application of IDPRs to MCGDM is demonstrated by investigating a manager selection problem for an automobile manufacturing company located in Wuhu, a city in Anhui province of China.

### Description of the manager selection problem

With the rapid development of economy and society, enterprises must strive to compete in global contexts. To achieve the high competitiveness, enterprises require various kinds of resources, such as hardware sources, software sources, and human resources. Among these, human resources are the most important ones to attain competitive advantages for enterprises. Talent loss and disruption will inevitably result in the loss of enterprises and negatively influence their operation, especially for the enterprises with high personnel demand and high personnel mobility, such as the automobile industry. To avoid the abnormal operation of the enterprises due to talent loss and disruption, appropriate human resources must be ensured when some positions are unoccupied. How to select the appropriate human resource for a specific position is a challenge for such enterprises.

In practice, how to select an appropriate human resource can be considered as a complex MCGDM problem which plays a key role in the long-term development of enterprises [47,48]. Such a problem can be analyzed by the MCGDM process as described in Fig 1 when the preferences of decision makers are characterized by IDPRs. To demonstrate the analysis process, a manager selection problem is investigated for an automobile manufacturing enterprise located in Wuhu, a city in Anhui province of China.

Like other automobile enterprises, the enterprise located in Wuhu faces the problem of personnel loss. This will negatively contribute to the normal operation and the sustainable development of the enterprise. To avoid the occurrence of such a situation, the enterprise must select appropriate human resources for unoccupied positions. In the following, the selection of a senior manager of the quality control center is analyzed to demonstrate the application of IDPRs to MCGDM.

For some reasons, there is a vacant position for the senior manager of the quality control center in the enterprise. To occupy the position, the enterprise identifies five candidates represented by *a*_*l*_(*l* = 1,…,5) and aims to select the most appropriate one. The five ones are quality system manager, purchasing quality manager, product quality manager, after-sales quality manager, and manufacturing quality engineer. They have worked for the enterprise beyond eight years. The deputy general manager is pointed by the board of directors to act as a facilitator for the selection of the appropriate manager. Four decision makers are invited to conduct the selection process, including the director of human resources department, the director of sales department, the director of the quality management center, and the director of the manufacturing center. The four decision makers have cooperated with the five candidates for at least three years. Meanwhile, the detailed materials about the five candidates are given to the four decision makers to help them implement the selection process.

Ten criteria are identified as *e*_*i*_(*i* = 1,…,10) through a discussion among the facilitator and the four decision makers to help compare the five candidates. They are educational background, sense of responsibility, creativity and innovation, socializing, strategic planning management, leadership, team management, professional expertise, performance, and potential. The descriptions of the ten criteria are shown in Table A of Appendix B in S1 File.

After studying the documents concerning the ten criteria, the facilitator uses a way discussed in [45] to generate the relative weights of the ten criteria, i.e., *w* = (0.07, 0.06, 0.09, 0.12, 0.15, 0.15, 0.05, 0.08, 0.1, 0.08). The detailed process for generating criterion weights is presented in Appendix B in S1 File. In a similar way, the facilitator assigns the relative weights of the four decision makers on each criterion according to their domain knowledge and experience, which are presented in Table B of Appendix B in S1 File. The four decision makers use a set of grades to compare the five candidates, i.e., *Ω* = {*H*_1_,*H*_2_,…,*H*_11_} = {absolutely weaker, very much weaker, much weaker, moderately weaker, marginally weaker, indifferent, marginally stronger, moderately stronger, much stronger, very much stronger, absolutely stronger}. Through a probability assignment approach [49], the facilitator sets *s*(*H*_*n*_)(*n* = 1,…,11) to be (−1, −0.8, −0.6, −0.4, −0.2, 0, 0.2, 0.4, 0.6, 0.8, 1) with the integration of the opinions of the four decision makers.

### Analysis of the manager selection problem

Through analyzing the sub-perspectives of each criterion that are presented in Table A of Appendix B in S1 File and the detailed materials about the five candidates, the four decision makers provide the IDPRs between neighboring candidates in terms of their own expertise and experience. The relevant IDPRs are presented in Table C of Appendix B in S1 File. Through using the ER algorithm and the relative weights of the four decision makers, the IDPRs between neighboring candidates on each criterion provided by the four decision makers are aggregated as the group IDPRs, which are presented in Table D of Appendix B in S1 File. Solving the optimization models shown in Eqs (22)–(25) and (26)–(29) with the group IDPRs and *s*(*H*_*n*_) transforms the group IDPRs into their score intervals, which are presented in Table 1.

**Table 1:** Score intervals of the group IDPRs between neighboring candidates in the manager selection problem.

Criteria	*s*(*e*_*i*_(*a*_12_))	*s*(*e*_*i*_(*a*_23_))	*s*(*e*_*i*_(*a*_34_))	*s*(*e*_*i*_(*a*_45_))
*e*_1_	[−0.5854, −0.2631]	[−0.5048, −0.2578]	[0.3328, 0.5984]	[−0.5692, −0.2930]
*e*_2_	[0.3697, 0.5865]	[0.1632, 0.3934]	[−0.4644, −0.2630]	[0.1361, 0.3649]
*e*_3_	[0.1482, 0.4263]	[−0.0106, 0.2235]	[−0.0848, 0.1577]	[−0.4205, −0.1908]
*e*_4_	[0.3613, 0.6760]	[0.1269, 0.3420]	[−0.4263, −0.1835]	[0.2796, 0.5577]
*e*_5_	[−0.5204, −0.2268]	[−0.4693, −0.2239]	[−0.5243, −0.2054]	[−0.5631, −0.3514]
*e*_6_	[0.0414, 0.4196]	[0.2360, 0.5350]	[0.2728, 0.5048]	[−0.5886, −0.3221]
*e*_7_	[−0.3124, 0.0553]	[−0.1638, 0.0340]	[0.2493, 0.4484]	[0.1545, 0.4546]
*e*_8_	[−0.3904, −0.1686]	[−0.5341, −0.2827]	[−0.4323, −0.2480]	[−0.6968, −0.4475]
*e*_9_	[−0.2628, −0.0280]	[0.2739, −0.4703]	[−0.6071, −0.3375]	[0.0122, 0.3364]
*e*_10_	[0.3212, 0.5170]	[−0.4596, 0.2093]	[0.0068, 0.2278]	[−0.3703, 0.1116]

With the consideration of the score intervals presented in Table 1, the facilitator selects a specific pair of candidates *a*_1_ and *a*_3_ and requires the four decision makers to provide the IDPRs on each criterion to learn the parameters *a* and *b* of the group for the additive consistency presented in Definitions 9 and 10. The provided IDPRs are presented in Table C of Appendix B in S1 File and the group IDPRs are obtained through the ER algorithm and the relative weights of the four decision makers and shown in Table D of Appendix B in S1 File. From solving the optimization models shown in Eqs (22)–(25) and (26)–(29), *s*(*e*_*i*_(*a*_13_))(*i* = 1,…,10) can be obtained as {[−0.8564, −0.5771], [0.5712, 0.7562], [0.2365, 0.4365], [0.5687, 0.7594], [−0.7844, −0.6025], [0.646, 0.8278], [−0.465, −0.2113], [−0.7849, −0.5859], [0.0464, 0.262], [0.1039, 0.3302]}. An optimization model shown in Eqs (41)–(53) with *s*(*e*_*i*_(*a*_12_)), *s*(*e*_*i*_(*a*_23_)), and *s*(*e*_*i*_(*a*_13_)) is then solved to obtain that *a* = −2.3333 and *b* = 0.3752.

After the parameters of the group for the additive consistency presented in Definitions 9 and 10 are determined, the group IDPRs on each criterion are aggregated by using the ER algorithm and the relative weights of the ten criteria to generate *r*(*a*_*l*(*l*+1)_)(*l* = 1,…,4), as presented in Table 2.

**Table 2:** Aggregated IDPRs between neighboring candidates in the manager selection problem.

Candidates	*r*(*a*_*l*(*l*+1)_)(*l* = 1,…,4)
*a*_12_	{(*H*_2_, [0.0215, 0323]), (*H*_3_, [0.0393, 0.0576]), (*H*_4_, [0.1612, 0.2305]), (*H*_5_, [0.1119, 0.1758]), (*H*_6_, [0.0761, 0.1036]), (*H*_7_, [0.1021, 0.1543]), (*H*_8_, [0.12, 0.1807]), (*H*_9_, [0.1359, 0.1898]), (*H*_10_, [0.0385, 0.0701]), (*Ω*, [0, 0.1211])}
*a*_23_	{(*H*_2_, [0.0122, 0.0155]), (*H*_3_, [0.0626, 0.093]), (*H*_4_, [0.1999, 0.2604]), (*H*_5_, [0.0694, 0.1202]), (*H*_6_, [0.0783, 0.1073]), (*H*_7_, [0.1768, 0.2443]), (*H*_8_, [0.1565, 0.2244]), (*H*_9_, [0.0517, 0.0803]), (*H*_10_, [0.0265, 0.037]), (*Ω*, [0, 0.1015])}
*a*_34_	{(*H*_2_, [0.0015, 0.0035]), (*H*_3_, [0.1618, 0.2229]), (*H*_4_, [0.1714, 0.253]), (*H*_5_, [0.1167, 0.17]), (*H*_6_, [0.0973, 0.127]), (*H*_7_, [0.0646, 0.1028]), (*H*_8_, [0.1472, 0.1949]), (*H*_9_, [0.0528, 0.0822]), (*H*_10_, [0.0182, 0.0271]), (*Ω*, [0, 0.1046])}
*a*_45_	{(*H*_2_, [0.0672, 0.0911]), (*H*_3_, [0.1785, 0.2515]), (*H*_4_, [0.1998, 0.3021]), (*H*_5_, [0.0989, 0.1389]), (*H*_6_, [0.0244, 0.0421]), (*H*_7_, [0.1098, 0.1604]), (*H*_8_, [0.0808, 0.1301]), (*H*_9_, [0.0463, 0.618]), (*H*_10_, [0.0117, 0.0222]), (*Ω*, [0, 0.1086])}

After *r*(*a*_*l*(*l*+1)_)(*l* = 1,…,4) shown in Table 2 is transformed into *s*(*a*_*l*(*l*+1)_), *s*(*a*_*lm*_)(*l*,*m* ∈ {1,…,5}, *m* > *l* + 1) can be obtained from *s*(*a*_*l*(*l*+1)_) through the additive consistency presented in Definitions 9 and 10 with the resulting parameters *a* and *b*. Then, *s*(*a*_*lm*_)(*l*,*m* ∈ {1,…,5}, *m* > *l* + 1) is obtained from *s*(*a*_*lm*_)(*l*,*m* ∈ {1,…,5}, *m* > *l* + 1) in accordance with Proposition 1. The relevant results are shown in Table 3.

**Table 3:** Score intervals of the IDPRs between any two candidates in the manager selection problem.

Candidates	*a*_1_	*a*_2_	*a*_3_	*a*_4_	*a*_5_
*a*_1_	[0, 0]	[−0.1048, 0.224]	[−0.2512, 0.3869]	[−0.4782, 0.461]	[−0.7426, 0.2722]
*a*_2_	[−0.224, 0.1048]	[0, 0]	[−0.1357, 0.1485]	[−0.3651, 0.2241]	[−0.6657, 0.125]
*a*_3_	[−0.3869, 0.2512]	[−0.1485, 0.1357]	[0, 0]	[−0.2152, 0.0674]	[−0.5462, 0.0273]
*a*_4_	[−0.461, 0.4782]	[−0.2241, 0.3651]	[−0.0674, 0.2152]	[0, 0]	[−0.3269, −0.0237]
*a*_5_	[−0.2722, 0.7426]	[−0.125, 0.6657]	[−0.0273, 0.5462]	[0.0237, 0.3269]	[0, 0]

From the results in Table 3, a possibility matrix *P* = (*p*(*s*(*a*_*lm*_) ≥ *s*(*a*_*ml*_)))_5×5_ is obtained by using Definition 7 and shown in Table 4. Then, a ranking order of the five candidates with possibility degrees is generated by using Definition 8 as $a_{5}\;\overset{1}{≻}a_{4}\;\overset{0.5181}{≻}a_{1}\;\overset{0.7968}{≻}a_{2}\overset{0.544}{≻}\;a_{3}$, which is a solution to the problem. The most appropriate candidate is *a*_5_, namely the manufacturing quality engineer.

**Table 4:** Possibility matrix of the five candidates in the manager selection problem.

Candidates		*a*_1_		*a*_2_		*a*_3_		*a*_4_		*a*_5_	
*a*_1_		0.5		0.7968		0.69		0.4819		0.1439	
*a*_2_		0.2032		0.5		0.544		0.2894		0.05	
*a*_3_		0.31		0.4273		0.5		0.1139		0.0045	
*a*_4_		0.5181		0.704		0.8861		0.5		0.00	
*a*_5_		0.8561		0.9500		0.9955		1		0.5	

### Analysis of the parameters associated with the additive consistency in the manager selection problem

In the process of solving the manager selection problem, only group parameters associated with the additive consistency are learned from the preferences of the four decision makers. In fact, individual parameters associated with the additive consistency can also be learned from the preferences of the four decision makers. In the following, we analyze the relationship between individual parameters and group parameters through the data in the manager selection problem.

As demonstrated in Eqs (54)–(58), multiple sets of individual parameters and group parameters are suggested to be used to analyze their relationship. To conduct the analysis, *λ* is changed from 0.15 to -0.15 with a step of 0.005 to result in 61 sets of *s*(*H*_*n*_). Through using the 61 sets of *s*(*H*_*n*_), the optimization model shown in Eqs (41)–(53) with *s*(*e*_*i*_(*a*_12_)), *s*(*e*_*i*_(*a*_23_)), and *s*(*e*_*i*_(*a*_13_)) is solved 61 times to generate 61 sets of *δ*_*j*_(*δ*_*j*_ ∈ {*a*_*j*_, *b*_*j*_}) and *δ*(*δ* ∈ {*a*, *b*}), which are presented in Tables E and F of Appendix B in S1 File, respectively and plotted in Figures C and D of Appendix B in S1 File, respectively. Given *u*(*α*_*j*_) and $v(\delta_{j}^{i})$, the optimization model shown in Eqs (54)–(58) with the 61 sets of *δ*_*j*_ and *δ* can be solved to obtain the minimum error between *δ* and the combination of *δ*_*j*_. Different sets of *u*(*α*_*j*_) and $v(\delta_{j}^{i})$ may generate different minimum errors. To make the minimum error as small as possible, a preferable set of *u*(*α*_*j*_) and $v(\delta_{j}^{i})$ should be identified. Through observing the movement of *δ*_*j*_ and *δ* shown in Figures C and D of Appendix B in S1 File, three typical elementary functions are selected to combine individual parameters *δ*_*j*_, including the exponential function, the logarithmic function, and the power function. With the consideration of the symmetry of *u*(*α*_*j*_) and $v(\delta_{j}^{i})$ in the function of combining individual parameters, as shown in Eq (55), six sets of *u*(*α*_*j*_) and $v(\delta_{j}^{i})$ are generated from the three elementary functions to perform the experiments with the 61 sets of *δ*_*j*_ and *δ*. The six sets of functions and the relevant optimization results are shown in Table 5.

**Table 5:** The six sets of functions for the least square method and their relevant optimization results.

Functions	Results
*$g_{1}=\delta_{1}^{}\cdot \alpha_{1}^{c}+\delta_{2}^{}\cdot \alpha_{2}^{c}+\delta_{3}^{}\cdot \alpha_{3}^{c}+\delta_{4}^{}\cdot \alpha_{4}^{c},(c>0)$*	*c* = 0.928; *θ* = 0.89; *α*_1_ = 0.1; *α*_2_ = 0.67; *α*_3_ = 0.1; *α*_4_ = 0.13
*g*_2_ = −*δ*_1_ *log*_*c*_*a*_1_ − *δ*_2_ *log*_*c*_*a*_2_ − *δ*_3_ *log*_*c*_*a*_3_ − *δ*_4_ *log*_*c*_*a*_4_, (*c* > 0)	*c* = 3500; *θ* = 1.12; *α*_1_ = 0.1; *α*_2_ = 0.1; *α*_3_ = 0.7; *α*_4_ = 0.1
*$g_{3}=\delta_{1}^{c}\cdot a_{1}+\delta_{2}^{c}\cdot a_{2}+\delta_{3}^{c}\cdot a_{3}+\delta_{4}^{c}\cdot a_{4},(c>0)$*	*c* = 3; *θ* = 34640.32; *α*_1_ = 0.1; *α*_2_ = 0.7; *α*_3_ = 0.1; *α*_4_ = 0.1
*$g_{4}=c^{\delta_{1}}\cdot a_{1}+c^{\delta_{2}}\cdot a_{2}+c^{\delta_{3}}\cdot a_{3}+c^{\delta_{4}}\cdot a_{4},(c>0)$*	*c* = 1000; *θ* = 269.45; *α*_1_ = 0.34; *α*_2_ = 0.1; *α*_3_ = 0.11; *α*_4_ = 0.45
*g*_5_ = (*δ*_1_ *a*_1_)^*c*^ + (*δ*_2_ *a*_2_)^*c*^ + (*δ*_3_ *a*_3_)^*c*^ + (*δ*_4_ *a*_4_)^*c*^, (*c* > 0)	*c* = 3; *θ* = 15.52; *α*_1_ = 0.13; *α*_2_ = 0.67; *α*_3_ = 0.1; *α*_4_ = 0.1
*$g_{6}=\delta_{1}^{}\cdot \;c^{\alpha_{1}}+\delta_{2}^{}\cdot c^{\alpha_{2}}+\delta_{3}^{}\cdot c^{\alpha_{3}}+\delta_{4}^{}\cdot c^{\alpha_{4}},(c>0)$*	*c* = 0.001; *θ* = 0.73; *α*_1_ = 0.18; *α*_2_ = 0.1; *α*_3_ = 0.49; *α*_4_ = 0.23

**Fig 2:**
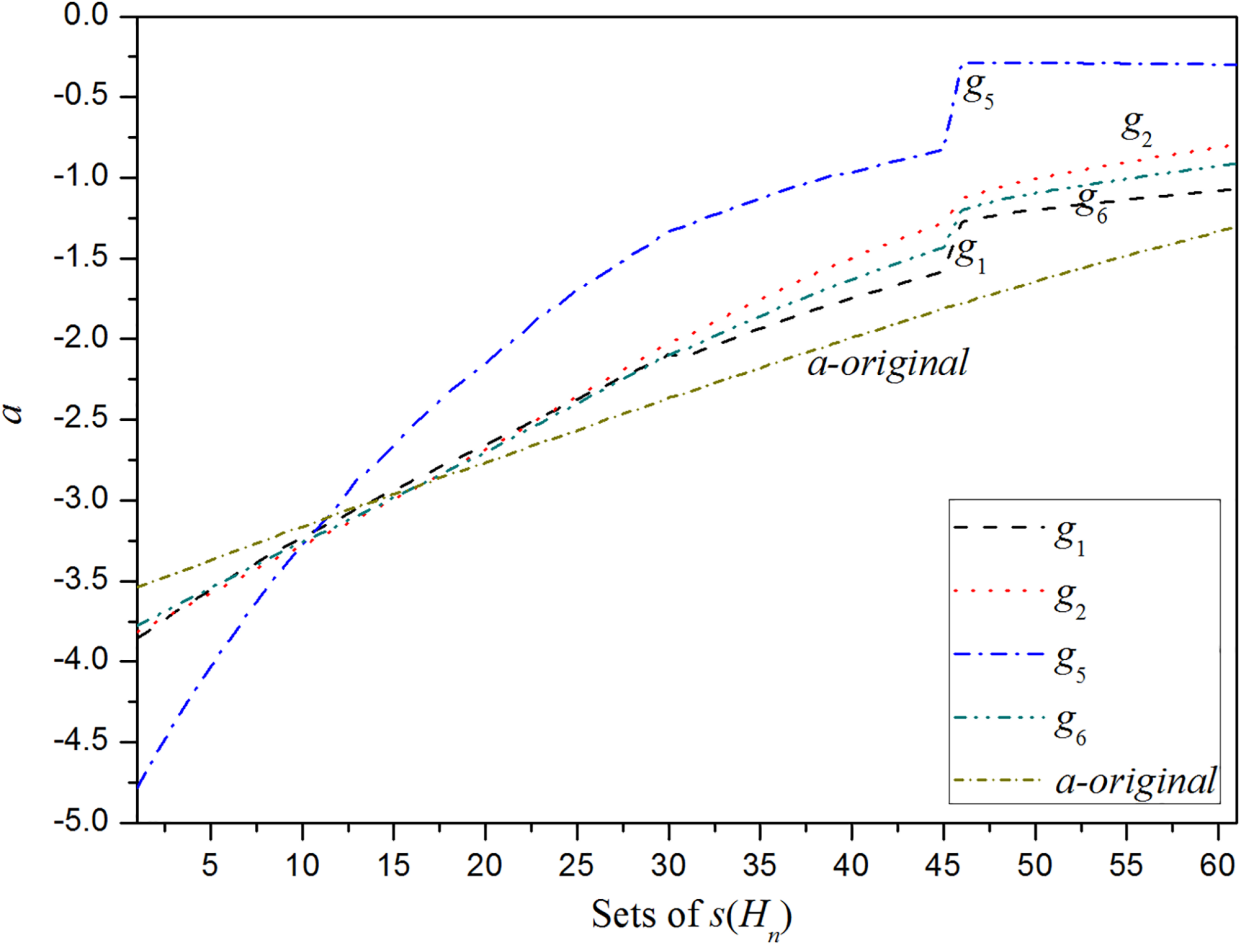
The comparison of the combinations of *a*_*j*_ generated by using the four sets of functions for the manager selection problem.

**Fig 3:**
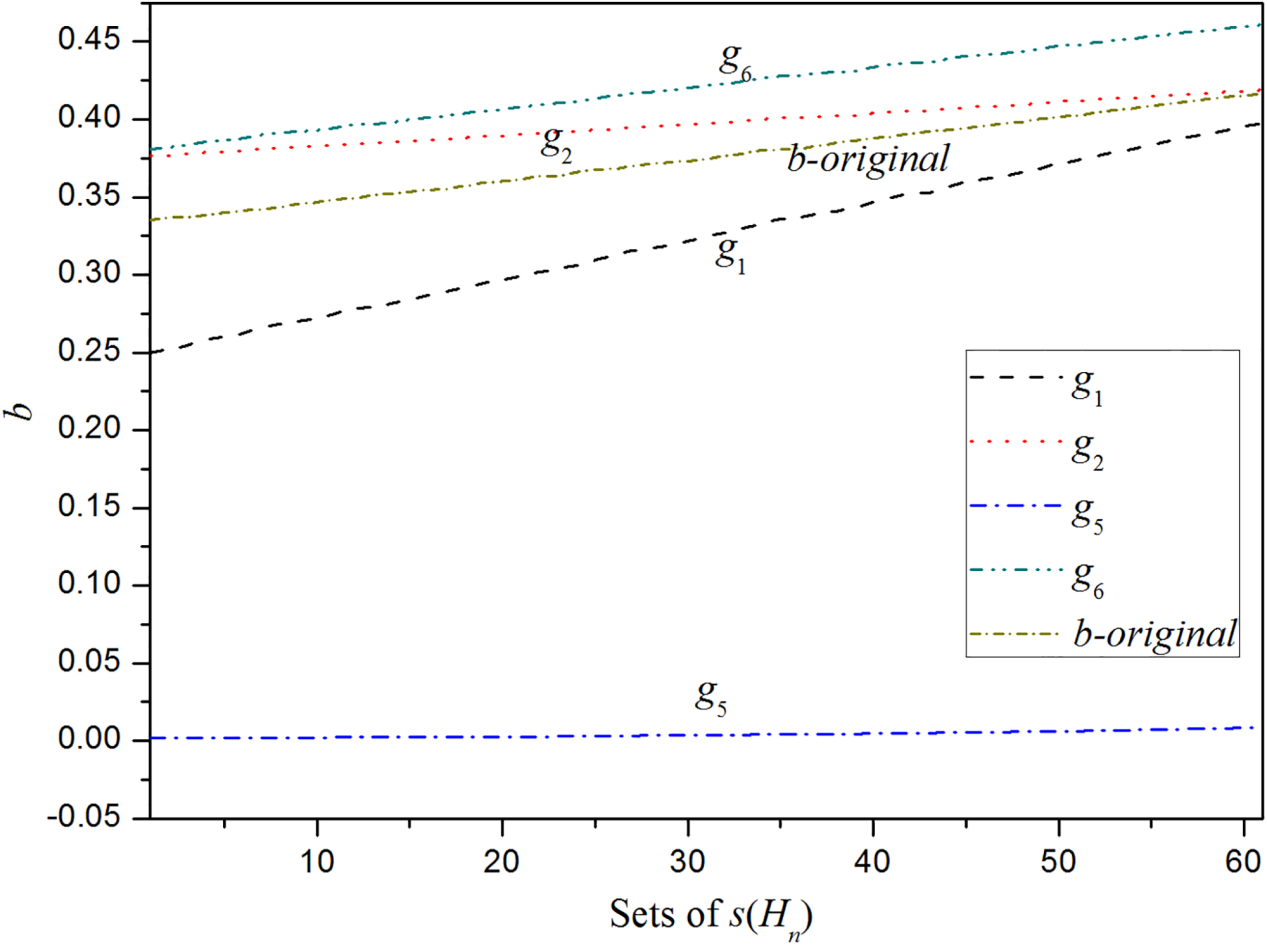
The comparison of the combinations of *b*_*j*_ generated by using the four sets of functions for the manager selection problem.

Table 5 shows that the third and fourth sets of *u*(*α*_*j*_) and $v(\delta_{j}^{i})$ result in significantly large minimum errors, which indicates the inferior performances of the two sets of functions to fit the relationship between *δ*_*j*_ and *δ*. To compare the performances of the other four sets of functions, the combinations of *a*_*j*_(*b*_*j*_) associated with the four sets of functions are calculated in accordance with the 61 sets of *s*(*H*_*n*_). The results and *a*(*b*) are plotted in Figs 2 and 3.

Figs 2 and 3 indicate that the tendency of the combinations of *δ*_*j*_ generated by using the first and sixth sets of functions is very close to the tendency of *δ*. It can also be found from Table 5 that the minimum errors generated by using the first and sixth sets of functions are less than those by using the second and fifth sets of functions. Specifically, although the minimum error generated by using the sixth set of functions is less than that by using the first set of functions, as shown in Table 5, the tendency of the combinations of *δ*_*j*_ generated by using the first set of functions is closer to the tendency of *δ*. From this perspective, the first set of functions is better than the sixth set of functions to fit the relationship between *δ*_*j*_ and *δ*. It is indicted from Table 5 that there is a nonlinear relationship between *δ*_*j*_ and *δ*. As the optimized parameter *c* of the function *u*(*α*_*j*_) is very close to 1, the nonlinear relationship revealed by the first set of functions is very close to a linear one.

## Conclusions

As an effective way to describe the relationship between two alternatives, DPR can characterize the preferred, non-preferred, indifferent, and uncertain degrees of one alternative over another. Although the DPR is flexible, it cannot handle some situations, such as the comparison between two radiologists from their examination reports, as presented in Introduction. To address such situations, this paper extends DPR to IDPR. To facilitate the comparison between IDPRs, the IDPRs are transformed into their score intervals through constructing two optimization models shown in Eqs (22)–(25) and (26)–(29). The additive consistency of the score intervals between alternatives is defined with two parameters to guarantee the rationality of the comparisons between alternatives. The two parameters are determined by the preferences of decision makers to ensure that the solution derived from the comparisons between alternatives is what is anticipated by the decision makers. IDPRs are then used to model and analyze MCGDM problems. To relieve the burden on decision makers, only the IDPRs between neighboring alternatives are required to be provided. The score intervals of the remaining IDPRs between non-neighboring alternatives are obtained from the score intervals of the provided IDPRs through the additive consistency with the two parameters derived from the preferences of the decision makers. A solution is generated by using the score intervals of the IDPRs between all alternatives. The application of IDPRs to MCGDM is demonstrated by analyzing a manager selection problem for an automobile manufacturing enterprise located in Wuhu, a city of Anhui province in China. In the application, it is highlighted that the cooperation between the candidates and the decision makers and the materials about the candidates aid the decision makers in providing the reliable IDPRs between the candidates. In particular, the relationship between the individual parameters of each decision maker and the group parameters for the defined additive consistency is analyzed in the context of the manager selection problem.

As shown in Illustrative example, a facilitator is included in the process of analyzing the manager selection problem. In the next step, we will investigate the application of IDPRs to MCGDM without a facilitator. In addition, we will also focus on group consensus in the application of IDPRs to MCGDM, in which the measurement of group consensus and the acceleration mechanism of group consensus convergence will be investigated.

## Supporting information

S1 File Supplementary material of “Interval-valued distributed preference relation and its application to group decision making”. Appendix A. (Figure A) The function *G*′ with respect to *x*. (Figure B) The function *K*′ with respect to *y*. Appendix B. (Table A) Explanation of the ten criteria in the manager selection problem. (Table B) Relative weights of the four decision makers on the ten criteria in the manager selection problem. (Table C) IDPRs between the neighboring candidates and the IDPRs between specific pair of candidates used to construct additive consistency in the manager selection problem. (Table D) Group IDPRs between the neighboring candidates and the group IDPRs between specific pair of candidates used to construct additive consistency in the manager selection problem. (Table E) Values of the parameter *a* for decision makers and the group with the variation in *s*(*H*_*n*_). (Figure C) Movement of the parameter *a* for decision makers and the group with the variation in *s*(*H*_*n*_). (Table F) Values of the parameter *b* for decision makers and the group with the variation in *s*(*H*_*n*_). (Figure D) Movement of the parameter *b* for decision makers and the group with the variation in *s*(*H*_*n*_).
(DOC)
