# Supplementary material for: Interval-valued distributed preference relation and its application to group decision making
Source: PLoS One. 2018 Jun 11;13(6):e0198393. doi: 10.1371/journal.pone.0198393 (PMC5995369; doi:10.1371/journal.pone.0198393)
Supplement: S1 File — Appendix A. (Figure A) The function G′ with respect to x. (Figure B) The function K′ with respect to y. Appendix B. (Table A) Explanation of the ten criteria in the manager selection problem. (Table B) Relative weights of the four decision makers on the ten criteria in the manager selection problem. (Table C) IDPRs between the neighboring candidates and the IDPRs between specific pair of candidates used to construct additive consistency in the manager selection problem. (Table D) Group IDPRs between the neighboring candidates and the group IDPRs between specific pair of candidates used to construct additive consistency in the manager selection problem. (Table E) Values of the parameter a for decision makers and the group with the variation in s(Hn). (Figure C) Movement of the parameter a for decision makers and the group with the variation in s(Hn). (Table F) Values of the parameter b for decision makers and the group with the variation in s(Hn). (Figure D) Movement of the parameter b for decision makers and the group with the variation in s(Hn). (DOC) [file pone.0198393.s001.doc]

**Supplementary material of “Interval-valued distributed preference relation and its application to group decision making”**

Yin Liu^1,2^, Chao Fu^1,2,*^, Min Xue^1,2^, Wenjun Chang^1,2^, Shanlin Yang^1,2^

^1^School of Management, Hefei University of Technology, Hefei, Box 270, Hefei 230009, Anhui, P.R. China

^2^Key Laboratory of Process Optimization and Intelligent Decision-making, Ministry of Education, Hefei 230009, Anhui, P.R. China

^*^Corresponding author. Tel: 0086-551-62904930; fax: 0086-551-62905263.

*E-mail address:* wls_fuchao@163.com (C. Fu).

**Appendix A**

## **Proof of Proposition 1**

**Proof of Proposition 1.** To facilitate analyzing the conclusions in Eqs. (30)-(31), the objective in Eq. (22) is rewritten as

$$\mathrm{MIN} s_{ij}=\sum_{n=1}^{(\text{N}+1)/2-1} m_{ij}(\text{H}_{n})\cdot\text{s}(\text{H}_{n})\cdot s(H_{n})+\sum_{n=(N+1)/2+1}^{N} m_{ij}(H_{n})\cdot s(H_{n})+m_{ij}(\text{Ω})\cdot s(H_{1})$$

According to Definitions 1 and 2, we have, $m_{ij}(\text{Ω})=m_{ji}(\text{Ω})$ and. From these facts, Eq. (1) can be transformed into

$$\mathrm{MIN} s_{ij}=-\sum_{n=(N+1)/2+1}^{N} m_{ji}(H_{n})\cdot s(H_{n})+\sum_{n=1}^{(N+1)/2-1} m_{ji}(H_{n})\cdot s(H_{n})+m_{ji}(\text{Ω})\cdot s(H_{N})$$

On the other hand, in accordance with the above facts, the objective in Eq. (26) can be transformed into

$$-\mathrm{MAX} s_{ji}=\sum_{n=(N+1)/2+1}^{N} m_{ij}(H_{n})\cdot s(H_{n})+\sum_{n=1}^{(N+1)/2-1} m_{ij}(H_{n})\cdot s(H_{n})+m_{ij}(\text{Ω})\cdot s(H_{N})$$

Eqs. (A.2) and (A.3) show that $\mathrm{MIN}s_{ij}=-\mathrm{MAX}s_{ji}$, which means that $s_{ij}^{-}+s_{ji}^{+}=0$. Similarly, $s_{ij}^{+}+s_{ji}^{-}=0$ can be verified. □

**Proof of Proposition 2**

**Proof of Proposition 2.** Suppose that $-1<x<0$ and $0<y<1$. Under the conditions, it can be obtained from $\min\{x,y\}\leq q(x,y)\leq\max\{x,y\}$ that $x\leq q(x,y)$ and$q(x,y)\leq y$. The inequalities of$x\leq q(x,y)$ and $q(x,y)\leq y$ can be transformed into $G=\frac{(1-b)\cdot(x+y)}{1-b\cdot xy}-x=$ $\frac{b\cdot yx^{2}-b\cdot x+(1-b)\cdot y}{1-b\cdot xy}\geq0$ and $K=\frac{(1-b)\cdot(x+y)}{1-b\cdot xy}-y=\frac{b\cdot xy^{2}-b\cdot y+(1-b)\cdot x}{1-b\cdot xy}\leq0$.

From$-1<x<0$, $0<y<1$, and$0\leq b\leq\text{ }1$, we can infer that $1-b\cdot xy>0$. In this situation, $G\geq\text{ }0$ and $K\leq0$ are equivalent to $\text{G}'=b\cdot yx^{2}-b\cdot x+(1-b)\cdot y\geq0$ and $\text{K}'\text{=}$ $b\cdot xy^{2}-b\cdot y+(1-b)\cdot x\leq0$. Let us focus on $\text{G}'\geq0$ first. Suppose that $\text{G}'$ is the function with respect to$x$, which is plotted in Figure A. From Figure A, it is shown that $\text{G}'\geq0$ holds when$-1<x<0$. As a result, $G\geq0$ is verified.

**Figure A.** The function $\text{G}'$ with respect to *x*.

Next, we prove that $\text{K}'\leq0$. The function $\text{K}'$ with respect to $y$ is plotted in Figure B. From this figure, it can be reasoned that $\text{K}'\leq0$ holds when$0<y<1$. As a result, $K\leq0$ is verified.

**Figure B.** The function $\text{K}'$ with respect to *y*.

Moreover, we can infer from $G\geq0$ that$(1-b)\cdot y\geq\text{ }0$, i.e., $0\leq1-b$. On the condition that $-1<x<0$ and$0<y<1$, $G\geq0$ requires that $0\leq b\leq1$. Similarly, it can be reasoned that $K\leq\text{ }0$ requires that $0\leq b\leq1$. Therefore, the parameter *b* is limited to $[0,\text{ }1]$.

Given $x$, the partial derivative of $q$ with respect to $y$ is calculated as

$$\frac{\partial q}{\partial y}=\frac{1}{(1-b\cdot xy)^{2}}\cdot\text{ }(1-b)\cdot(1-b\cdot xy)-(1-b)\cdot\text{ }(x+y)\cdot\text{ }(-b\cdot x)$$

$$=\frac{1}{(1-b\cdot xy)^{2}}\cdot(1-b)\cdot(1-b\cdot xy+b\cdot x(x+y))$$

$$=\frac{1}{(1-b\cdot xy)^{2}}\cdot(1-b)\cdot(1+b\cdot x^{2})$$

When$-1<x<0$, $0<y<1$, and$0\leq b\leq1$, $\frac{\partial q}{\partial y}\geq0$ holds. This indicates that $q(x,y_{1})>q(x,y_{2})$ when$y_{2}<y_{1}$. Eq. (39) is verified.

Eq. (40) can be similarly verified. □

**Proof of Proposition 3**

**Proof of Proposition 3.** Given $x$ and $y$, the partial derivative of $q$ with respect to $b$ is calculated as

$$\frac{\partial q}{\partial b}=\frac{1}{(1-b\cdot xy)^{2}}\cdot(-(x\text{ }+\text{ }y))\cdot\text{ }(1-b\cdot xy)-(1-b)\cdot\text{ }(x+y)\cdot\text{ }(-xy)$$

$$=\frac{1}{(1-b\cdot xy)^{2}}\cdot(x+y)\cdot((1-b)\cdot xy-1\text{ }+b\cdot xy)$$

$$=\frac{1}{(1-b\cdot xy)^{2}}\cdot(x+y)\cdot(xy-1)$$

If $x+y<\text{ }0$, we can infer from $xy-1<0$ that $\frac{\partial q}{\partial b}>0$. If $x+y>0$, we can infer that $\frac{\partial q}{\partial b}<0$. As a result, the conclusions in this proposition are verified. □

**Proof of Theorem 1**

**Proof of Theorem 1.** The given conditions indicate that $x<\text{ }0.5$ and $y>0.5$ or $x>0.5$ and $y<0.5$. Thus, it can be inferred from Proposition 2 that

$$g(t(x),t(y))=q(t(x),t(y))$$

$$=\frac{(1-b)\cdot(2(x-0.5) +2(y-0.5))}{1-b\cdot2(x-0.5)\cdot2(y-0.5)}$$

$=\frac{(1-b)\cdot(2x +2y-2)}{1-4b\cdot(x-0.5)\cdot(y-0.5)}$.

When $g(t(x),t(y)) +t(\mathrm{z})\text{ }=\text{ }0$, we can further reason that $t(\text{z})=\frac{-(1-b)\cdot(2x +2y-2)}{1-4b\cdot(x-0.5)\cdot(y-0.5)}$. Because$t(z)=2(z-0.5)$, it can be obtained that $z=\frac{-(1-b)\cdot(x +y-1)}{1-4b\cdot(x-0.5)\cdot(y-0.5)}+0.5$.

Suppose that $\Delta S=x+y+z-1.5$, then we have

$$\Delta S=x+y-1+\frac{-(1-b)\cdot(x +y-1)}{1-4b\cdot(x-0.5)\cdot(y-0.5)}$$

$=\text{ }(x+y-1)\cdot\frac{b-4b\cdot(x-0.5)\cdot(y-0.5)}{1-4b\cdot(x-0.5)\cdot(y-0.5)}$.

Due to the fact that $0\leq b\leq1$, when $x<0.5$ and $y>0.5$ or $x>0.5$ and$y<0.5$, $\Delta S$ may be greater than, equal to, or less than 0.

Therefore, under the given conditions $x+y+z=1.5$ can only be seen as a special case of$f(p(x),p(y))+p(z)=0$. □

# Appendix B

**Explanation of criteria and determination of criterion weights for the manager selection problem**

After studying the documents concerning the ten criteria, the facilitator uses the method discussed in (Ölçer and Odabaşi, 2005) to determine criterion weights *w*. Assume that $w=(w_{1},\ldots,w_{10})$ denotes the relative importance of the ten criteria that is not normalized. First of all, the facilitator selects the most important criterion, which is the fifth criterion, and specifies that *w*_5_ = 1. Then, the facilitator compares the other criteria with the fifth one to determine the relative importance of these criteria, which is . It is clear that max$\{w_{1},\text{ }\ldots,w_{10}\}=1$ and min$\{w_{1},\text{ }\ldots,w_{10}\}>0$. Finally,$w_{i}(i=\text{ }1,\text{ }\ldots,\text{ }10)$ is calculated as $w_{i}=\frac{w_{i}}{\sum_{j=1}^{10} w_{j}}$, which is $(w_{1},\text{ }\ldots,w_{10})=(0.07,\text{ }0.06,\text{ }0.09,\text{ }0.12,\text{ }0.15,\text{ }0.15,\text{ }0.05,\text{ }0.08,\text{ }0.1,\text{ }0.08)$.

The relevant explanation of the ten criteria is presented in Table A.

**Table A.** Explanation of the ten criteria in the manager selection problem.

| **Criteria** | **Explanation** |
| --- | --- |
| Personality ($e_{1}$)  0.07 | Employee’s education background and professional integrity. |
| Sense of responsibility ($e_{2}$)  0.06 | Employee’s capability of effectively and enthusiastically accomplishing assignments with the minimal supervision. |
| Learning and innovation ($e_{3}$)  0.09 | Employee’s capability of independently and quickly learning and grasping new knowledge and creatively accomplishing assignments. |
| Socializing ($e_{4}$)  0.12 | Employee’s capability of influencing others, such as establishing and maintaining positive and productive working relationship.  Employee’s communication skills, such as inspiring cooperation and mutual respect, accepting suggestions, and working with others effectively to achieve goals. |
| Management capabilities ($e_{5}$)  0.15 | Employee capability of playing multiple roles, such as dividing a project into subitems to facilitate completing the project, arranging time effectively to improve the efficiency. |
| Leadership ($e_{6}$)  0.15 | Employee’s capability of acting as a leader, such as understanding the enterprise’s culture and idea, following the enterprise’s development strategy, analyzing faults to make correct decisions, implementing correct decisions, organizing subordinates, and designing workflow to accomplish organizational goals. |
| Employing and educating personnel ($e_{7}$)  0.05 | Employee’s capability of evaluating subordinates and training the needed talents. |
| Expertise ($e_{8}$)  0.08 | Employee’s knowledge and experience associated with his or her position, including awareness, practices, manual skills, and techniques in relevant fields. |
| Performance ($e_{9}$)  0.1 | Employee’s capability of maintaining effective time management and meeting productivity standards. |
| Potential ($e_{10}$)  0.08 | Employee’s development path and development plan. Employee’s working experience in the relevant fields. |

**Determination of the relative weights of decision makers in the manager selection problem**

**Table B.** Relative weights of the four decision makers on the ten criteria in the manager selection problem.

| **Criteria** | ***t*_1_** | ***t*_2_** | ***t*_3_** | ***t*_4_** |
| --- | --- | --- | --- | --- |
| $e_{1}$ | 0.3 | 0.25 | 0.25 | 0.2 |
| $e_{2}$ | 0.25 | 0.35 | 0.2 | 0.2 |
| $e_{3}$ | 0.2 | 0.35 | 0.25 | 0.2 |
| $e_{4}$ | 0.2 | 0.3 | 0.25 | 0.25 |
| $e_{5}$ | 0.2 | 0.3 | 0.25 | 0.25 |
| $e_{6}$ | 0.35 | 0.25 | 0.15 | 0.25 |
| *e*_7_ | 0.25 | 0.35 | 0.25 | 0.15 |
| $e_{8}$ | 0.3 | 0.35 | 0.25 | 0.1 |
| $e_{9}$ | 0.25 | 0.35 | 0.25 | 0.15 |
| $e_{10}$ | 0.25 | 0.35 | 0.2 | 0.2 |

**Intermediate results in the manager selection problem**

**Table C.** IDPRs between the neighboring candidates and the IDPRs between specific pair of

candidates used to construct additive consistency in the manager selection problem.

| **Criteria** | $R(e_{i}(a_{\mathbf{12}}))$ | $R(e_{i}(a_{\mathbf{23}}))$ | $R(e_{i}(a_{\mathbf{34}}))$ | $R(e_{i}(a_{\mathbf{45}}))$ | $R(e_{i}(a_{\mathbf{13}}))$ |
| --- | --- | --- | --- | --- | --- |
| $e_{1}$ | *t*_1_: {(*H*_4_, [0.4, 0.5]),  (*H*_5_, [0.5, 0.6]),  (*Ω*, [0, 0.1])}  *t*_2_: {(*H*_3_, [0.6, 0.7]),  (*H*_4_, [0.2, 0.4]),  (*Ω*, [0, 0.2])}  *t*_3_: {(*H*_3_, [0.6, 0.8]),  (*H*_4_, [0.2, 0.3]),  (*Ω*, [0, 0.2])}  *t*_4_: {(*H*_2_, [0.5, 0.7]),  (*H*_3_, [0.3, 0.5]),  (*Ω*, [0, 0.2])} | *t*_1_: {(*H*_3_, [0.5, 0.6]),  (*H*_4_, [0.4, 0.5]),  (*Ω*, [0, 0.1])}  *t*_2_: {(*H*_2_, [0.8, 0.9]),  (*H*_3_, [0.1, 0.2]),  (*Ω*, [0, 0.1])}  *t*_3_: {(*H*_5_, [0.4, 0.5]),  (*H*_6_, [0.5, 0.6]),  (*Ω*, [0, 0.1])}  *t*_4_: {(*H*_4_, [0.3, 0.4]),  (*H*_5_, [0.5, 0.7]),  (*Ω*, [0, 0.2])} | *t*_1_: {(*H*_9_, [0.4, 0.5]),  (*H*_10_, [0.5, 0.6]),  (*Ω*, [0, 0.1])}  *t*_2_: {(*H*_8_, [0.7, 0.8]),  (*H*_9_, [0.2, 0.3]),  (*Ω*, [0, 0.1])}  *t*_3_: {(*H*_8_, [0.5, 0.7]),  (*H*_9_, [0.3, 0.4]),  (*Ω*, [0, 0.2])}  *t*_4_: {(*H*_7_, [0.1, 0.2]),  (*H*_8_, [0.5, 0.6]),  (*H*_9_, [0.2, 0.3]),  (*Ω*, [0, 0.2])} | *t*_1_: {(*H*_2_, [0.3, 0.4]),  (*H*_3_, [0.6, 0.7]),  (*Ω*, [0, 0.1])}  *t*_2_: {(*H*_3_, [0.7, 0.8]),  (*H*_4_, [0.2, 0.3]),  (*Ω*, [0, 0.1])}  *t*_3_: {(*H*_4_, [0.3, 0.5]),  (*H*_5_, [0.5, 0.6]),  (*Ω*, [0, 0.2])}  *t*_4_: {(*H*_4_, [0.6, 0.8]),  (*H*_5_, [0.2, 0.3]),  (*Ω*, [0, 0.2])} | *t*_1_: {(*H*_2_, [0.5, 0.7]),  (*H*_3_, [0.3, 0.5]),  (*Ω*, [0, 0.2])}  *t*_2_: {(*H*_1_, [0.6, 0.7]),  (*H*_2_, [0.2, 0.4]),  (*Ω*, [0, 0.2])}  *t*_3_: {(*H*_2_, [0.7, 0.8]),  (*H*_3_, [0.2, 0.3]),  (*Ω*, [0, 0.1])}  *t*_4_: {(*H*_1_, [0.6, 0.7]),  (*H*_2_, [0.3, 0.4]),  (*Ω*, [0, 0.1])} |
| $e_{2}$ | *t*_1_: {(*H*_8_, [0.7, 0.8]),  (*H*_9_, [0.2, 0.3]),  (*Ω*, [0, 0.1])}  *t*_2_: {(*H*_8_, [0.7, 0.8]),  (*H*_9_, [0.2, 0.3]),  (*Ω*, [0, 0.1])}  *t*_3_: {(*H*_9_, [0.5, 0.7]),  (*H*_10_, [0.3, 0.4]),  (*Ω*, [0, 0.2])}  *t*_4_: {(*H*_9_, [0.4, 0.5]),  (*H*_10_, [0.5, 0.6]),  (*Ω*, [0, 0.1])} | *t*_1_: {(*H*_7_, [0.4, 0.5]),  (*H*_8_, [0.5, 0.6]),  (*Ω*, [0, 0.1])}  *t*_2_: {(*H*_7_, [0.2, 0.3]),  (*H*_8_, [0.7, 0.8]),  (*Ω*, [0, 0.1])}  *t*_3_: {(*H*_7_, [0.6, 0.8]),  (*H*_8_, [0.2, 0.4]),  (*Ω*, [0, 0.2])}  *t*_4_: {(*H*_7_, [0.5, 0.7]),  (*H*_8_, [0.3, 0.4]),  (*Ω*, [0, 0.2])} | *t*_1_: {(*H*_4_, [0.5, 0.6]),  (*H*_5_, [0.4, 0.5]),  (*Ω*, [0, 0.1])}  *t*_2_: {(*H*_4_, [0.8, 0.9]),  (*H*_5_, [0.1, 0.2]),  (*Ω*, [0, 0.1])}  *t*_3_: {(*H*_3_, [0.4, 0.6]),  (*H*_4_, [0.4, 0.5]),  (*Ω*, [0, 0.2])}  *t*_4_: {(*H*_3_, [0.4, 0.5]),  (*H*_4_, [0.5, 0.7]),  (*Ω*, [0, 0.1])} | *t*_1_: {(*H*_7_, [0.6, 0.7]),  (*H*_8_, [0.3, 0.4]),  (*Ω*, [0, 0.1])}  *t*_2_: {(*H*_7_, [0.7, 0.8]),  (*H*_8_, [0.2, 0.3]),  (*Ω*, [0, 0.1])}  *t*_3_: {(*H*_7_, [0.2, 0.3]),  (*H*_8_, [0.6, 0.8]),  (*Ω*, [0, 0.2])}  *t*_4_: {(*H*_7_, [0.3, 0.4]),  (*H*_8_, [0.5, 0.7]),  (*Ω*, [0, 0.2])} | *t*_1_: {(*H*_9_, [0.7, 0.8]),  (*H*_10_, [0.2, 0.3]),  (*Ω*, [0, 0.1])}  *t*_2_: {(*H*_9_, [0.8, 0.9]),  (*H*_10_, [0.1, 0.2]),  (*Ω*, [0, 0.1])}  *t*_3_: {(*H*_10_, [0.6, 0.7]),  (*H*_11_, [0.3, 0.4]),  (*Ω*, [0, 0.1])}  *t*_4_: {(*H*_10_, [0.3, 0.4]),  (*H*_11_, [0.6, 0.7]),  (*Ω*, [0, 0.1])} |
| $e_{3}$ | *t*_1_: {(*H*_7_, [0.6, 0.7]),  (*H*_8_, [0.2, 0.4]),  (*Ω*, [0, 0.2])}  *t*_2_: {(*H*_7_, [0.4, 0.5]),  (*H*_8_, [0.4, 0.6]),  (*Ω*, [0, 0.2])}  *t*_3_: {(*H*_7_, [0.5, 0.7]),  (*H*_8_, [0.4, 0.5]),  (*Ω*, [0, 0.1])}  *t*_4_: {(*H*_8_, [0.3, 0.4]),  (*H*_9_, [0.6, 0.7]),  (*Ω*, [0, 0.1])} | *t*_1_: {(*H*_6_, [0.4, 0.5]),  (*H*_7_, [0.5, 0.6]),  (*Ω*, [0, 0.1])}  *t*_2_: {(*H*_7_, [0.3, 0.4]),  (*H*_8_, [0.6, 0.7]),  (*Ω*, [0, 0.1])}  *t*_3_: {(*H*_5_, [0.4, 0.5]),  (*H*_6_, [0.5, 0.6]),  (*Ω*, [0, 0.1])}  *t*_4_: {(*H*_5_, [0.1, 0.2]),  (*H*_6_, [0.7, 0.9]),  (*Ω*, [0, 0.2])} | *t*_1_: {(*H*_5_, [0.4, 0.6]),  (*H*_6_, [0.5, 0.6]),  (*Ω*, [0, 0.1])}  *t*_2_: {(*H*_6_, [0.5, 0.6]),  (*H*_7_, [0.4, 0.5]),  (*Ω*, [0, 0.1])}  *t*_3_: {(*H*_6_, [0.6, 0.7]),  (*H*_7_, [0.2, 0.4]),  (*Ω*, [0, 0.2])}  *t*_4_: {(*H*_6_, [0.5, 0.6]),  (*H*_7_, [0.3, 0.5]),  (*Ω*, [0, 0.2])} | *t*_1_: {(*H*_2_, [0.3, 0.4]),  (*H*_3_, [0.5, 0.6]),  (*H*_4_, [0.1, 0.2]),  (*Ω*, [0, 0.1])}  *t*_2_: {(*H*_4_, [0.2, 0.3]),  (*H*_5_, [0.7, 0.8]),  (*Ω*, [0, 0.1])}  *t*_3_: {(*H*_4_, [0.1, 0.2]),  (*H*_5_, [0.7, 0.9]),  (*H*_6_, [0.1, 0.2]),  (*Ω*, [0, 0.1])}  *t*_4_: {(*H*_3_, [0.3, 0.4]),  (*H*_4_, [0.5, 0.7]),  (*Ω*, [0, 0.2])} | *t*_1_: {(*H*_8_, [0.6, 0.7]),  (*H*_9_, [0.3, 0.4]),  (*Ω*, [0, 0.1])}  *t*_2_: {(*H*_8_, [0.2, 0.3]),  (*H*_9_, [0.7, 0.8]),  (*Ω*, [0, 0.1])}  *t*_3_: {(*H*_6_, [0.7, 0.8]),  (*H*_7_, [0.2, 0.3]),  (*Ω*, [0, 0.1])}  *t*_4_: {(*H*_7_, [0.5, 0.6]),  (*H*_8_, [0.4, 0.5]),  (*Ω*, [0, 0.1])} |
| $e_{4}$ | *t*_1_: {(*H*_8_, [0, 0.2]),  (*H*_9_, [0.7, 0.8]),  (*H*_10_, [0.2, 0.3]),  (*Ω*, [0, 0.1])}  *t*_2_: {(*H*_9_, [0.5, 0.6]),  (*H*_10_, [0.3, 0.5]),  (*Ω*, [0, 0.2])}  *t*_3_: {(*H*_8_, [0.5, 0.7]),  (*H*_9_, [0.3, 0.4]),  (*Ω*, [0, 0.2])}  *t*_4_: {(*H*_8_, [0.1, 0.2]),  (*H*_9_, [0.5, 0.7]),  (*H*_10_, [0.2, 0.3]),  (*Ω*, [0, 0.2])} | *t*_1_: {(*H*_7_, [0.7, 0.9]),  (*H*_8_, [0.1, 0.2]),  (*Ω*, [0, 0.2])}  *t*_2_: {(*H*_7_, [0.1, 0.2]),  (*H*_8_, [0.8, 0.9]),  (*Ω*, [0, 0.1])}  *t*_3_: {(*H*_6_, [0.4, 0.5]),  (*H*_7_, [0.5, 0.6]),  (*Ω*, [0, 0.1])}  *t*_4_: {(*H*_7_, [0.5, 0.7]),  (*H*_8_, [0.4, 0.5]),  (*Ω*, [0, 0.1])} | *t*_1_: {(*H*_4_, [0.3, 0.4]),  (*H*_5_, [0.6, 0.7]),  (*Ω*, [0, 0.1])}  *t*_2_: {(*H*_3_, [0.8, 0.9]),  (*H*_4_, [0.1, 0.2]),  (*Ω*, [0, 0.1])}  *t*_3_: {(*H*_5_, [0.7, 0.8]),  (*H*_6_, [0.2, 0.4]),  (*Ω*, [0, 0.1])}  *t*_4_: {(*H*_4_, [0.5, 0.7]),  (*H*_5_, [0.3, 0.5]),  (*Ω*, [0, 0.2])} | *t*_1_: {(*H*_9_, [0.4, 0.5]),  (*H*_10_, [0.4, 0.6]),  (*Ω*, [0, 0.2])}  *t*_2_: {(*H*_9_, [0.8, 0.9]),  (*H*_10_, [0.1, 0.2]),  (*Ω*, [0, 0.1])}  *t*_3_: {(*H*_7_, [0.4, 0.5]),  (*H*_8_, [0.4, 0.6]),  (*Ω*, [0, 0.2])}  *t*_4_: {(*H*_7_, [0.4, 0.5]),  (*H*_8_, [0.5, 0.6]),  (*Ω*, [0, 0.1])} | *t*_1_: {(*H*_9_, [0.1, 0.2]),  (*H*_10_, [0.8, 0.9]),  (*Ω*, [0, 0.1])}  *t*_2_: {(*H*_10_, [0.7, 0.8]),  (*H*_11_, [0.2, 0.3]),  (*Ω*, [0, 0.1])}  *t*_3_: {(*H*_9_, [0.2, 0.3]),  (*H*_10_, [0.7, 0.8]),  (*Ω*, [0, 0.1])}  *t*_4_: {(*H*_7_, [0, 0.2]),  (*H*_8_, [0.5, 0.6]),  (*H*_9_, [0.4, 0.5]),  (*Ω*, [0, 0.1])} |
| $e_{5}$ | *t*_1_: {(*H*_4_, [0.5, 0.6]),  (*H*_5_, [04, 0.5]),  (*Ω*, [0, 0.1])}  *t*_2_: {(*H*_4_, [0.6, 0.8]),  (*H*_5_, [0.2, 0.3]),  (*Ω*, [0, 0.2])}  *t*_3_: {(*H*_2_, [0.5, 0.7]),  (*H*_3_, [0.4, 0.6]),  (*Ω*, [0, 0.1])}  *t*_4_: {(*H*_4_, [0.6, 0.8]),  (*H*_5_, [0.2, 0.4]),  (*Ω*, [0, 0.2])} | *t*_1_: {(*H*_4_, [0.7, 0.8]),  (*H*_5_, [0.2, 0.3]),  (*Ω*, [0, 0.1])}  *t*_2_: {(*H*_3_, [0.3, 0.4]),  (*H*_4_, [0.6, 0.8]),  (*Ω*, [0, 0.1])}  *t*_3_: {(*H*_4_, [0.6, 0.8]),  (*H*_5_, [0.2, 0.4]),  (*Ω*, [0, 0.2])}  *t*_4_: {(*H*_4_, [0.6, 0.8]),  (*H*_5_, [0.2, 0.4]),  (*Ω*, [0, 0.2])} | *t*_1_: {(*H*_3_, [0.6, 0.8]),  (*H*_4_, [0.2, 0.4]),  (*Ω*, [0, 0.2])}  *t*_2_: {(*H*_4_, [0.3, 0.4]),  (*H*_5_, [0.5, 0.7]),  (*Ω*, [0, 0.2])}  *t*_3_: {(*H*_3_, [0.3, 0.4]),  (*H*_4_, [0.3, 0.4]),  (*H*_5_, [0.3, 0.4]),  (*Ω*, [0, 0.1])}  *t*_4_: {(*H*_3_, [0.6, 0.8]),  (*H*_4_, [0.2, 0.3]),  (*Ω*, [0, 0.2])} | *t*_1_: {(*H*_3_, [0.8, 0.9]),  (*H*_4_, [0.1, 0.2]),  (*Ω*, [0, 0.1])}  *t*_2_: {(*H*_3_, [0.7, 0.8]),  (*H*_4_, [0.2, 0.3]),  (*Ω*, [0, 0.1])}  *t*_3_: {(*H*_3_, [0.1, 0.2]),  (*H*_4_, [0.8, 0.9]),  (*Ω*, [0, 0.1])}  *t*_4_: {(*H*_3_, [0.3, 0.4]),  (*H*_4_, [0.5, 0.7]),  (*Ω*, [0, 0.2])} | *t*_1_: {(*H*_2_, [0.5, 0.6]),  (*H*_3_, [0.4, 0.5]),  (*Ω*, [0, 0.1])}  *t*_2_: {(*H*_2_, [0.4, 0.5]),  (*H*_3_, [0.5, 0.6]),  (*Ω*, [0, 0.1])}  *t*_3_: {(*H*_1_, [0.6, 0.7]),  (*H*_2_, [0.3, 0.4]),  (*Ω*, [0, 0.1])}  *t*_4_: {(*H*_2_, [0.5, 0.6]),  (*H*_3_, [0.4, 0.5]),  (*Ω*, [0, 0.1])} |
| $e_{6}$ | *t*_1_: {(*H*_6_, [0.6, 0.7]),  (*H*_7_, [0.2, 0.4]),  (*Ω*, [0, 0.2])}  *t*_2_: {(*H*_7_, [0.6, 0.8]),  (*H*_8_, [0.2, 0.3]),  (*Ω*, [0, 0.2])}  *t*_3_: {(*H*_7_, [0.5, 0.6]),  (*H*_8_, [0.4, 0.6]),  (*Ω*, [0, 0.1])}  *t*_4_: {(*H*_9_, [0.6, 0.7]),  (*H*_10_, [0.2, 0.4]),  (*Ω*, [0, 0.2])} | *t*_1_: {(*H*_8_, [0.5, 0.6]),  (*H*_9_, [0.3, 0.5]),  (*Ω*, [0, 0.2])}  *t*_2_: {(*H*_9_, [0.3, 0.4]),  (*H*_10_, [0.6, 0.7]),  (*Ω*, [0, 0.1])}  *t*_3_: {(*H*_7_, [0.6, 0.8]),  (*H*_8_, [0.2, 0.4]),  (*Ω*, [0, 0.2])}  *t*_4_: {(*H*_7_, [0.8, 0.9]),  (*H*_8_, [0.1, 0.2]),  (*Ω*, [0, 0.1])} | *t*_1_: {(*H*_8_, [0.8, 0.9]),  (*H*_9_, [0.1, 0.3]),  (*Ω*, [0, 0.1])}  *t*_2_: {(*H*_7_, [0.3, 0.4]),  (*H*_8_, [0.5, 0.7]),  (*Ω*, [0, 0.2])}  *t*_3_: {(*H*_7_, [0.5, 0.6]),  (*H*_8_, [0.4, 0.5]),  (*Ω*, [0, 0.1])}  *t*_4_: {(*H*_8_, [0.1, 0.2]),  (*H*_9_, [0.5, 0.7]),  (*H*_10_, [0.3, 0.5]),  (*Ω*, [0, 0.1])} | *t*_1_: {(*H*_2_, [0.4, 0.5]),  (*H*_3_, [0.5, 0.6]),  (*Ω*, [0, 0.1])}  *t*_2_: {(*H*_4_, [0.8, 0.9]),  (*H*_5_, [0.1, 0.2]),  (*Ω*, [0, 0.1])}  *t*_3_: {(*H*_4_, [0.5, 0.7]),  (*H*_5_, [0.3, 0.4]),  (*Ω*, [0, 0.2])}  *t*_4_: {(*H*_3_, [0.4, 0.6]),  (*H*_4_, [0.4, 0.6]),  (*Ω*, [0, 0.2])} | *t*_1_: {(*H*_9_, [0.4, 0.5]),  (*H*_10_, [0.5, 0.6]),  (*Ω*, [0, 0.1])}  *t*_2_: {(*H*_10_, [0.3, 0.4]),  (*H*_11_, [0.6, 0.7]),  (*Ω*, [0, 0.1])}  *t*_3_: {(*H*_9_, [0.3, 0.4]),  (*H*_10_, [0.6, 0.7]),  (*Ω*, [0, 0.1])}  *t*_4_: {(*H*_9_, [0.1, 0.2]),  (*H*_10_, [0.4, 0.6]),  (*H*_11_, [0.4, 0.5]),  (*Ω*, [0, 0.1])} |
| $e_{7}$ | *t*_1_: {(*H*_4_, [0.3, 0.5]),  (*H*_5_, [0.5, 0.6]),  (*Ω*, [0, 0.2])}  *t*_2_: {(*H*_4_, [0.2, 0.3]),  (*H*_5_, [0.6, 0.8]),  (*Ω*, [0, 0.2])}  *t*_3_: {(*H*_6_, [0.5, 0.6]),  (*H*_7_, [0.3, 0.5]),  (*Ω*, [0, 0.2])}  *t*_4_: {(*H*_5_, [0.3, 0.4]),  (*H*_6_, [0.6, 0.7]),  (*Ω*, [0, 0.1])} | *t*_1_: {(*H*_5_, [0.4, 0.5]),  (*H*_6_, [0.5, 0.6]),  (*Ω*, [0, 0.1])}  *t*_2_: {(*H*_5_, [0.3, 0.4]),  (*H*_6_, [0.6, 0.7]),  (*Ω*, [0, 0.2])}  *t*_3_: {(*H*_5_, [0.1, 0.2]),  (*H*_6_, [0.7, 0.9]),  (*H*_7_, [0.1, 0.2]),  (*Ω*, [0, 0.1])}  *t*_4_: {(*H*_4_, [0.1, 0.2]),  (*H*_5_, [0.7, 0.9]),  (*Ω*, [0, 0.2])} | *t*_1_: {(*H*_7_, [0.4, 0.5]),  (*H*_8_, [0.5, 0.6]),  (*Ω*, [0, 0.1])}  *t*_2_: {(*H*_8_, [0.7, 0.8]),  (*H*_9_, [0.2, 0.3]),  (*Ω*, [0, 0.1])}  *t*_3_: {(*H*_7_, [0.5, 0.6]),  (*H*_8_, [0.4, 0.6]),  (*Ω*, [0, 0.1])}  *t*_4_: {(*H*_3_, [0.3, 0.4]),  (*H*_4_, [0.5, 0.7]),  (*Ω*, [0, 0.2])} | *t*_1_: {(*H*_8_, [0.3, 0.5]),  (*H*_9_, [0.5, 0.6]),  (*Ω*, [0, 0.2])}  *t*_2_: {(*H*_7_, [0.8, 0.9]),  (*H*_8_, [0.1, 0.2]),  (*Ω*, [0, 0.1])}  *t*_3_: {(*H*_7_, [0.3, 0.5]),  (*H*_8_, [0.5, 0.6]),  (*Ω*, [0, 0.2])}  *t*_4_: {(*H*_8_, [0.3, 0.5]),  (*H*_9_, [0.5, 0.7]),  (*Ω*, [0, 0.2])} | *t*_1_: {(*H*_2_, [0.3, 0.4]),  (*H*_3_, [0.5, 0.6]),  (*Ω*, [0, 0.2])}  *t*_2_: {(*H*_3_, [0.4, 0.5]),  (*H*_4_, [0.5, 0.6]),  (*Ω*, [0, 0.1])}  *t*_3_: {(*H*_6_, [0.1, 0.2]),  (*H*_7_, [0.8, 0.9]),  (*Ω*, [0, 0.1])}  *t*_4_: {(*H*_3_, [0.5, 0.6]),  (*H*_4_, [0.4, 0.5]),  (*Ω*, [0, 0.1])} |
| $e_{8}$ | *t*_1_: {(*H*_4_, [0.6, 0.7]),  (*H*_5_, [0.3,0.4]),  (*Ω*, [0, 0.1])}  *t*_2_: {(*H*_4_, [0.7, 0.8]),  (*H*_5_, [0.2, 0.3]),  (*Ω*, [0, 0.1])}  *t*_3_: {(*H*_5_, [0.5, 0.7]),  (*H*_6_, [0.3, 0.4]),  (*Ω*, [0, 0.2])}  *t*_4_: {(*H*_3_, [0.3, 0.5]),  (*H*_4_, [0.6, 0.8]),  (*Ω*, [0, 0.1])} | *t*_1_: {(*H*_3_, [0.4, 0.6]),  (*H*_4_, [0.4, 0.5]),  (*Ω*, [0, 0.2])}  *t*_2_: {(*H*_3_, [0.7, 0.8]),  (*H*_4_, [0.2, 0.4]),  (*Ω*, [0, 0.1])}  *t*_3_: {(*H*_4_, [0.5, 0.6]),  (*H*_5_, [0.4, 0.5]),  (*Ω*, [0, 0.1])}  *t*_4_: {(*H*_4_, [0.6, 0.8]),  (*H*_5_, [0.2, 0.4]),  (*Ω*, [0, 0.2])} | *t*_1_: {(*H*_3_, [0.4, 0.6]),  (*H*_4_, [0.5, 0.6]),  (*Ω*, [0, 0.1])}  *t*_2_: {(*H*_4_, [0.4, 0.5]),  (*H*_5_, [0.5, 0.6]),  (*Ω*, [0, 0.1])}  *t*_3_: {(*H*_4_, [0.4, 0.5]),  (*H*_5_, [0.5, 0.7]),  (*Ω*, [0, 0.1])}  *t*_4_: {(*H*_2_, [0.1, 0.2]),  (*H*_3_, [0.5, 0.6]),  (*H*_4_, [0.3, 0.5]),  (*Ω*, [0, 0.1])} | *t*_1_: {(*H*_2_, [0.6, 0.7]),  (*H*_3_, [0.3, 0.4]),  (*Ω*, [0, 0.1])}  *t*_2_: {(*H*_2_, [0.7, 0.8]),  (*H*_3_, [0.2, 0.3]),  (*Ω*, [0, 0.1])}  *t*_3_: {(*H*_4_, [0.6, 0.8]),  (*H*_5_, [0.2, 0.4]),  (*Ω*, [0, 0.2])}  *t*_4_: {(*H*_3_, [0.3, 0.4]),  (*H*_4_, [0.5, 0.7]),  (*Ω*, [0, 0.2])} | *t*_1_: {(*H*_2_, [0.7, 0.8]),  (*H*_3_, [0.2, 0.3]),  (*Ω*, [0, 0.1])}  *t*_2_: {(*H*_1_, [0.6, 0.7]),  (*H*_2_, [0.3, 0.4]),  (*Ω*, [0, 0.1])}  *t*_3_: {(*H*_3_, [0.4, 0.5]),  (*H*_4_, [0.5, 0.6]),  (*Ω*, [0, 0.1])}  *t*_4_: {(*H*_2_, [0.4, 0.5]),  (*H*_3_, [0.5, 0.6]),  (*Ω*, [0, 0.1])} |
| $e_{9}$ | *t*_1_: {(*H*_4_, [0.4, 0.5]),  (*H*_5_, [0.5, 0.6]),  (*Ω*, [0, 0.1])}  *t*_2_: {(*H*_5_, [0.3, 0.4]),  (*H*_6_, [0.6, 0.7]),  (*Ω*, [0, 0.1])}  *t*_3_: {(*H*_4_, [0.5, 0.7]),  (*H*_5_, [0.3, 0.5]),  (*Ω*, [0, 0.2])}  *t*_4_: {(*H*_6_, [0.6, 0.7]),  (*H*_7_, [0.3, 0.5]),  (*Ω*, [0, 0.1])} | *t*_1_: {(*H*_8_, [0.2, 0.3]),  (*H*_9_, [0.4, 0.5]),  (*H*_10_, [0.3, 0.4]),  (*Ω*, [0, 0.1])}  *t*_2_: {(*H*_7_, [0.7, 0.8]),  (*H*_8_, [0.2, 0.3]),  (*Ω*, [0, 0.1])}  *t*_3_: {(*H*_8_, [0.3, 0.4]),  (*H*_9_, [0.6, 0.7]),  (*Ω*, [0, 0.1])}  *t*_4_: {(*H*_7_, [0.6, 0.7]),  (*H*_8_, [0.3, 0.4]),  (*Ω*, [0, 0.1])} | *t*_1_: {(*H*_3_, [0.6, 0.7]),  (*H*_4_, [0.3, 0.4]),  (*Ω*, [0, 0.1])}  *t*_2_: {(*H*_3_, [0.7, 0.8]),  (*H*_4_, [0.1, 0.3]),  (*Ω*, [0, 0.2])}  *t*_3_: {(*H*_3_, [0.3, 0.4]),  (*H*_4_, [0.5, 0.6]),  (*Ω*, [0, 0.2])}  *t*_4_: {(*H*_2_, [0.1, 0.2]),  (*H*_3_, [0.5, 0.6]),  (*H*_4_, [0.3, 0.5]),  (*Ω*, [0, 0.1])} | *t*_1_: {(*H*_7_, [0.4, 0.5]),  (*H*_8_, [0.5, 0.6]),  (*Ω*, [0, 0.1])}  *t*_2_: {(*H*_6_, [0.6, 0.8]),  (*H*_7_, [0.2, 0.4]),  (*Ω*, [0, 0.2])}  *t*_3_: {(*H*_7_, [0.5, 0.7]),  (*H*_8_, [0.3, 0.5]),  (*Ω*, [0, 0.2])}  *t*_4_: {(*H*_7_, [0.5, 0.7]),  (*H*_8_, [0.3, 0.5]),  (*Ω*, [0, 0.2])} | *t*_1_: {(*H*_5_, [0.4, 0.5]),  (*H*_6_, [0.5, 0.6]),  (*Ω*, [0, 0.1])}  *t*_2_: {(*H*_6_, [0.2, 0.4]),  (*H*_7_, [0.6, 0.8]),  (*Ω*, [0, 0.2])}  *t*_3_: {(*H*_7_, [0.5, 0.6]),  (*H*_8_, [0.4, 0.5]),  (*Ω*, [0, 0.1])}  *t*_4_: {(*H*_8_, [0.6, 0.7]),  (*H*_9_, [0.3, 0.4]),  (*Ω*, [0, 0.1])} |
| $e_{10}$ | *t*_1_: {(*H*_7_, [0.7, 0.8]),  (*H*_8_, [0.2, 0.3]),  (*Ω*, [0, 0.1])}  *t*_2_: {(*H*_8_, [0.7, 0.8]),  (*H*_9_, [0.2, 0.4]),  (*Ω*, [0, 0.1])}  *t*_3_: {(*H*_9_, [0.5, 0.7]),  (*H*_10_, [0.4, 0.6]),  (*Ω*, [0, 0.1])}  *t*_4_: {(*H*_8_, [0.3, 0.4]),  (*H*_9_, [0.6, 0.7]),  (*Ω*, [0, 0.1])} | *t*_1_: {(*H*_3_, [0.4, 0.5]),  (*H*_4_, [0.5, 0.6]),  (*Ω*, [0, 0.1])}  *t*_2_: {(*H*_4_, [0.8, 0.9]),  (*H*_5_, [0.1, 0.2]),  (*Ω*, [0, 0.2])}  *t*_3_: {(*H*_5_, [0.4, 0.5]),  (*H*_6_, [0.4, 0.6]),  (*Ω*, [0, 0.2])}  *t*_4_: {(*H*_3_, [0.3, 0.5]),  (*H*_4_, [0.5, 0.6]),  (*Ω*, [0, 0.2])} | *t*_1_: {(*H*_6_, [0.6, 0.7]),  (*H*_7_, [0.3,0.4]),  (*Ω*, [0, 0.1])}  *t*_2_: {(*H*_6_, [0.8, 0.9]),  (*H*_7_, [0.1, 0.2]),  (*Ω*, [0, 0.2])}  *t*_3_: {(*H*_8_, [0.4, 0.5]),  (*H*_9_, [0.5, 0.6]),  (*Ω*, [0, 0.1])}  *t*_4_: {(*H*_6_, [0.3, 0.5]),  (*H*_7_, [0.5, 0.7]),  (*Ω*, [0, 0.2])} | *t*_1_: {(*H*_4_, [0.2, 0.3]),  (*H*_5_, [0.5,0.7]),  (*H*_6_, [0.1, 0.2]),  (*Ω*, [0, 0.2])}  *t*_2_: {(*H*_4_, [0.3, 0.4]),  (*H*_5_, [0.6, 0.7]),  (*Ω*, [0, 0.1])}  *t*_3_: {(*H*_3_, [0.2, 0.4]),  (*H*_4_, [0.6, 0.8]),  (*Ω*, [0, 0.2])}  *t*_4_: {(*H*_4_, [0.1, 0.2]),  (*H*_5_, [0.5, 0.6]),  (*H*_6_, [0.2, 0.3]),  (*Ω*, [0, 0.2])} | *t*_1_: {(*H*_6_, [0.6, 0.7]),  (*H*_7_, [0.3,0.4]),  (*Ω*, [0, 0.1])}  *t*_2_: {(*H*_7_, [0.5, 0.6]),  (*H*_8_, [0.4, 0.5]),  (*Ω*, [0, 0.1])}  *t*_3_: {(*H*_8_, [0.5, 0.6]),  (*H*_9_, [0.4, 0.5]),  (*Ω*, [0, 0.1])}  *t*_4_: {(*H*_6_, [0.3, 0.5]),  (*H*_7_, [0.5, 0.7]),  (*Ω*, [0, 0.2])} |

**Table D.** Group IDPRs between the neighboring candidates and the group IDPRs between specific pair of candidates used to construct additive consistency in the manager selection problem.

| **A** | | | | |
| --- | --- | --- | --- | --- |
| **Criteria** | $R(e_{i}(a_{\mathbf{12}}))$ | $R(e_{i}(a_{\mathbf{23}}))$ | | $R(e_{i}(a_{\mathbf{34}}))$ |
| *e*_1_ | {(*H*_2_, [0.0804, 0.1279]),  (*H*_3_, [0.3670, 0.5075]),  (*H*_4_, [0.2189, 0.3499]),  (*H*_5_, [0.1374, 0.1874]),  (*Ω*, [0, 0.1465])} | {(*H*_2_, [0.1859, 0.2249]),  (*H*_3_, [0.1785, 0.2496]),  (*H*_4_, [0.1820, 0.2450]),  (*H*_5_, [0.1929, 0.2719]),  (*H*_6_, [0.1161, 0.1499]),  (*Ω*, [0, 0.1097])} | | {(*H*_7_, [0.0156, 0.0348]),  (*H*_8_, [0.3993, 0.5214]),  (*H*_9_, [0.2844, 0.4069]),  (*H*_10_, [0.1337, 0.1796]),  (*Ω*, [0, 0.1219])} |
| *e*_2_ | {(*H*_8_, [0.4373, 0.5287]),  (*H*_9_, [0.2937, 0.4315]),  (*H*_10_, [0.1307, 0.1773]),  (*Ω*, [0, 0.1009])} | {(*H*_7_, [0.3609, 0.5283]),  (*H*_8_, [0.4717, 0.6248]),  (*Ω*, [0, 0.1093])} | | {(*H*_3_, [0.1202, 0.1851]),  (*H*_4_, [0.6442, 0.7530]),  (*H*_5_, [0.1119, 0.1808]),  (*Ω*, [0, 0.0949])} |
| *e*_3_ | {(*H*_7_, [0.3826, 0.4949]),  (*H*_8_, [0.3473, 0.5264]),  (*H*_9_, [0.0902, 0.1209]),  (*Ω*, [0, 0.1311])} | {(*H*_5_, [0.1062, 0.1574]),  (*H*_6_, [0.3392, 0.4446]),  (*H*_7_, [0.2023, 0.2752]),  (*H*_8_, [0.2085, 0.2647]),  (*Ω*, [0, 0.1056])} | | {(*H*_5_, [0.0575, 0.0819]),  (*H*_6_, [0.5548, 0.6765]),  (*H*_7_, [0.2309, 0.3719]),  (*Ω*, [0, 0.1146])} |
| *e*_4_ | {(*H*_8_, [0.1214, 0.2302]),  (*H*_9_, [0.5251, 0.6714]),  (*H*_10_, [0.1583, 0.2821]),  (*Ω*, [0, 0.1279])} | {(*H*_6_, [0.0819, 0.1138]),  (*H*_7_, [0.4263, 0.5690]),  (*H*_8_, [0.3481, 0.4532]),  (*Ω*, [0, 0.1000])} | | {(*H*_3_, [0.2223, 0.2763]),  (*H*_4_, [0.2057, 0.3232]),  (*H*_5_, [0.3763, 0.4980]),  (*H*_6_, [0.0435, 0.0719]),  (*Ω*, [0, 0.1101]) |
| *e*_5_ | {(*H*_2_, [0.1039, 0.1432]),  (*H*_3_, [0.0831, 0.1193]),  (*H*_4_, [0.4585, 0.6158]),  (*H*_5_, [0.1752, 0.2925]),  (*Ω*, [0, 0.1338])} | {(*H*_3_, [0.0701, 0.1090]),  (*H*_4_, [0.6705, 0.8230]),  (*H*_5_, [0.1066, 0.2302]),  (*Ω*, [0, 0.1152])} | | {(*H*_3_, [0.3340, 0.4829]),  (*H*_4_, [0.2525, 0.3947]),  (*H*_5_, [0.2119, 0.3253]),  (*Ω*, [0, 0.1489])} |
| *e*_6_ | {(*H*_6_, [0.2083, 0.2695]),  (*H*_7_, [0.3070, 0.4746]),  (*H*_8_, [0.0946, 0.1444]),  (*H*_9_, [0.1267, 0.1734]),  (*H*_10_, [0.0422, 0.0991]),  (*Ω*, [0, 0.1676])} | {(*H*_2_, [0.0606, 0.0928]),  (*H*_3_, [0.3225, 0.4100]),  (*H*_4_, [0.4019, 0.5530]),  (*H*_5_, [0.0431, 0.0976]),  (*Ω*, [0, 0.1291])} | | {(*H*_7_, [0.1193, 0.1674]),  (*H*_8_, [0.5394, 0.6813]),  (*H*_9_, [0.1389, 0.2159]),  (*H*_10_, [0.0602, 0.0905]),  (*Ω*, [0, 0.1056])} |
| *e*_7_ | {(*H*_4_, [0.1399, 0.2397]),  (*H*_5_, [0.3788, 0.5491]),  (*H*_6_, [0.1823, 0.2488]),  (*H*_7_, [0.0613, 0.1202]),  (*Ω*, [0, 0.1627]) | {(*H*_4_, [0.0100, 0.0224]),  (*H*_5_, [0.3074, 0.4408]),  (*H*_6_, [0.5293, 0.6429]),  (*H*_7_, [0.0192, 0.0424]),  (*Ω*, [0, 0.0911])} | | {(*H*_7_, [0.1905, 0.2553]),  (*H*_8_, [0.5635, 0.6776]),  (*H*_9_, [0.1209, 0.1930]),  (*Ω*, [0, 0.0932])} |
| *e*_8_ | {(*H*_3_, [0.0198, 0.0292]),  (*H*_4_, [0.5069, 0.6127]),  (*H*_5_, [0.2717, 0.4068]),  (*H*_6_, [0.0572, 0.0857]),  (*Ω*, [0, 0.10029])} | {(*H*_3_, [0.3695, 0.4959]),  (*H*_4_, [0.3755, 0.5082]),  (*H*_5_, [0.0939, 0.1452]),  (*Ω*, [0, 0.1169])} | | {(*H*_2_, [0.0066, 0.0146]),  (*H*_3_, [0.1408, 0.1902]),  (*H*_4_, [0.4480, 0.5645]),  (*H*_5_, [0.2874, 0.3687]),  (*Ω*, [0, 0.0850])} |
| *e*_9_ | {(*H*_4_, [0.2059, 0.2963]),  (*H*_5_, [0.3207, 0.4515]),  (*H*_6_, [0.2952, 0.3712]),  (*H*_7_, [0.0335, 0.0495]),  (*Ω*, [0, 0.1086])} | {(*H*_7_, [0.3380, 0.4113]),  (*H*_8_, [0.2433, 0.3590]),  (*H*_9_, [0.2339, 0.2994]),  (*H*_10_, [0.0642, 0.0927]),  (*Ω*, [0, 0.0890])} | | {(*H*_2_, [0.0099, 0.0232]),  (*H*_3_, [0.5704, 0.6950]),  (*H*_4_, [0.2459, 0.4003]),  (*Ω*, [0, 0.1290])} |
| *e*_10_ | {(*H*_7_, [0.1499, 0.1861]),  (*H*_8_, [0.3804, 0.4824]),  (*H*_9_, [0.2832, 0.3789]),  (*H*_10_, [0.0642, 0.0876]),  (*Ω*, [0, 0.0888])} | {(*H*_3_, [0.1357, 0.2077]),  (*H*_4_, [0.5585, 0.6602]),  (*H*_5_, [0.0968, 0.1621]),  (*H*_6_, [0.0598, 0.1019]),  (*Ω*, [0, 0.1149])} | | {(*H*_6_, [0.5384, 0.6592]),  (*H*_7_, [0.1884,0.3036]),  (*H*_8_, [0.0607, 0.0852]),  (*H*_9_, [0.0758, 0.1022]),  (*Ω*, [0, 0.1007])} |
| **B** | | | | |
| **Criteria** | $R(e_{i}(a_{\mathbf{45}}))$ | | $R\left(e_{i}\left(a_{\mathbf{13}}\right)\right)$ | |
| $e_{1}$ | {(*H*_2_, [0.0841, 0.1233]), (*H*_3_, [0.3664, 0.4532]), (*H*_4_, [0.2336, 0.3681]), (*H*_5_, [0.1474, 0.2031]), (*Ω*, [0, 0.1256])} | | {(*H*_1_, [0.2322, 0.3041]), (*H*_2_, [0.4690, 0.6433]), (*H*_3_, [0.1245, 0.2253]), (*Ω*, [0, 0.1310])} | |
| $e_{2}$ | {(*H*_7_, [0.5026, 0.6367]), (*H*_8_, [0.3348, 0.4974]), (*Ω*, [0, 0.1091])} | | {(*H*_9_, [0.4852, 0.5711]), (*H*_10_, [0.2484, 0.3648]), (*H*_11_, [0.1490, 0.1971]), (*Ω*, [0, 0.0863])} | |
| $e_{3}$ | {(*H*_2_, [0.0479, 0.0698]), (*H*_3_, [0.1344, 0.1816]), (*H*_4_, [0.2068, 0.3422]), (*H*_5_, [0.4515, 0.5401]), (*H*_6_, [0.0213, 0.0463]), (*Ω*, [0, 0.1032])} | | {(*H*_6_, [0.1533, 0.1899]), (*H*_7_, [0.1334, 0.1867]), (*H*_8_, [0.2720, 0.3639]), (*H*_9_, [0.3195, 0.3963]), (*Ω*, [0, 0.0906])} | |
| $e_{4}$ | {(*H*_7_, [0.1928, 0.2571]), (*H*_8_, [0.2187, 0.3128]), (*H*_9_, [0.3237, 0.3988]), (*H*_10_, [0.0993, 0.1782]), (*Ω*, [0, 0.1276])} | | {(*H*_7_, [0, 0.022]), (*H*_8_, [0.1018, 0.1342]), (*H*_9_, [0.1669, 0.2472]), (*H*_10_, [0.5640, 0.6569]), (*H*_11_, [0.0529, 0.1034]), (*Ω*, [0, 0.0839])} | |
| $e_{5}$ | {(*H*_3_, [0.4621, 0.5945]), (*H*_4_, [0.3860, 0.5379]), (*Ω*, [0, 0.0809])} | | {(*H*_1_, [0.1227, 0.1565]), (*H*_2_, [0.4390, 0.5575]), (*H*_3_, [0.3186, 0.4161]), (*Ω*, [0, 0.0840])} | |
| $e_{6}$ | {(*H*_2_, [0.1345, 0.1867]), (*H*_3_, [0.2784, 0.3916]), (*H*_4_, [0.3647, 0.4955]), (*H*_5_, [0.0564, 0.0982]), (*Ω*, [0, 0.1198])} | | {(*H*_9_, [0.1956, 0.2846]), (*H*_10_, [0.4695, 0.5859]), (*H*_11_, [0.2185, 0.2843]), (*Ω*, [0, 0.0846])} | |
| $e_{7}$ | {(*H*_7_, [0.3360, 0.4848]), (*H*_8_, [0.2753, 0.4379]), (*H*_9_, [0.1698, 0.2402]), (*Ω*, [0, 0.1198])} | | {(*H*_2_, [0.0637, 0.0930]), (*H*_3_, [0.3628, 0.4565]), (*H*_4_, [0.2357, 0.3042]), (*H*_6_, [0.0215, 0.0473]), (*H*_7_, [0.1721, 0.2130]), (*Ω*, [0, 0.1105])} | |
| $e_{8}$ | {(*H*_2_, [0.4636, 0.5555]), (*H*_3_, [0.1842, 0.2731]), (*H*_4_, [0.1627, 0.2445]), (*H*_5_, [0.0401, 0.0901]), (*Ω*, [0, 0.1141])} | | {(*H*_1_, [0.2048, 0.2563]), (*H*_2_, [0.3764, 0.4714]), (*H*_3_, [0.1917, 0.2706]), (*H*_4_, [0.1052, 0.1378]), (*Ω*, [0, 0.0899])} | |
| $e_{9}$ | {(*H*_6_, [0.1927, 0.2953]), (*H*_7_, [0.3836, 0.5886]), (*H*_8_, [0.2143, 0.3511]), (*Ω*, [0, 0.1503])} | | {(*H*_5_, [0.0866, 0.1169]), (*H*_6_, [0.1921, 0.3106]), (*H*_7_, [0.3598, 0.4829]), (*H*_8_, [0.1670, 0.2161]), (*H*_9_, [0.0347, 0.0498]), (*Ω*, [0, 0.0898])} | |
| $e_{10}$ | {(*H*_3_, [0.0294, 0.0669]), (*H*_4_, [0.2803, 0.4262]), (*H*_5_, [0.4553, 0.5919]), (*H*_6_, [0.0514, 0.0965]), (*Ω*, [0, 0.0994])} | | {(*H*_6_, [0.1865, 0.2646]), (*H*_7_, [0.3748, 0.4956]), (*H*_8_, [0.2365, 0.3117]), (*H*_9_, [0.0635, 0.0877]), (*Ω*, [0, 0.1037])} | |

**Table E. Values of the parameter *a* for decision makers and the group with the variation in $s(H_{n})$.**

| ***λ*** | ***a*** | ***a*_1_** | ***a*_2_** | ***a*_3_** | ***a*_4_** |
| --- | --- | --- | --- | --- | --- |
| | 0.15 | | --- | | 0.145 | | 0.14 | | 0.135 | | 0.13 | | 0.125 | | 0.12 | | 0.115 | | 0.11 | | 0.105 | | 0.1 | | 0.095 | | 0.09 | | 0.085 | | 0.08 | | 0.075 | | 0.07 | | 0.065 | | 0.06 | | 0.055 | | 0.05 | | 0.045 | | 0.04 | | 0.035 | | 0.03 | | 0.025 | | 0.02 | | 0.015 | | 0.01 | | 0.005 | | 0 | | -0.005 | | -0.01 | | -0.015 | | -0.02 | | -0.025 | | -0.03 | | -0.035 | | -0.04 | | -0.045 | | -0.05 | | -0.055 | | -0.06 | | -0.065 | | -0.07 | | -0.075 | | -0.08 | | -0.085 | | -0.09 | | -0.095 | | -0.1 | | -0.105 | | -0.11 | | -0.115 | | -0.12 | | -0.125 | | -0.13 | | -0.135 | | -0.14 | | -0.145 | | -0.15 | | | -3.5383 | | --- | | -3.4955 | | -3.4570 | | -3.4143 | | -3.3742 | | -3.3253 | | -3.2881 | | -3.2451 | | -3.1996 | | -3.1652 | | -3.1242 | | -3.0774 | | -3.0386 | | -3.0044 | | -2.9601 | | -2.9253 | | -2.8843 | | -2.8377 | | -2.8047 | | -2.7665 | | -2.7258 | | -2.6853 | | -2.6404 | | -2.6074 | | -2.5679 | | -2.5218 | | -2.4815 | | -2.4484 | | -2.4106 | | -2.3648 | | -2.3333 | | -2.2909 | | -2.2519 | | -2.2172 | | -2.1798 | | -2.1382 | | -2.0991 | | -2.0671 | | -2.0275 | | -1.9897 | | -1.9568 | | -1.9187 | | -1.8850 | | -1.8471 | | -1.8076 | | -1.7785 | | -1.7432 | | -1.7113 | | -1.6759 | | -1.6444 | | -1.6108 | | -1.5789 | | -1.5469 | | -1.5091 | | -1.4847 | | -1.4523 | | -1.4233 | | -1.3939 | | -1.3645 | | -1.3324 | | -1.3035 | | | -3.4953 | | --- | | -3.4257 | | -3.3583 | | -3.2885 | | -3.2233 | | -3.1566 | | -3.0855 | | -3.0242 | | -2.9553 | | -2.8969 | | -2.8398 | | -2.7812 | | -2.7227 | | -2.6641 | | -2.5984 | | -2.5495 | | -2.4881 | | -2.4335 | | -2.3798 | | -2.3220 | | -2.2695 | | -2.2151 | | -2.1606 | | -2.1055 | | -2.0516 | | -2.0004 | | -1.9471 | | -1.8973 | | -1.8465 | | -1.7917 | | -1.7456 | | -1.6960 | | -1.6466 | | -1.5914 | | -1.5469 | | -1.5018 | | -1.4506 | | -1.4085 | | -1.3633 | | -1.3190 | | -1.2692 | | -1.2289 | | -1.1837 | | -1.1380 | | -1.0985 | | -1.0567 | | -1.0164 | | -0.9760 | | -0.9315 | | -0.8962 | | -0.8549 | | -0.8194 | | -0.7810 | | -0.7441 | | -0.7071 | | -0.6654 | | -0.6322 | | -0.5964 | | -0.5638 | | -0.5248 | | -0.4909 | | | -2.3719 | | --- | | -2.3430 | | -2.3161 | | -2.2885 | | -2.2606 | | -2.2335 | | -2.2048 | | -2.1772 | | -2.1505 | | -2.1234 | | -2.0983 | | -2.0717 | | -2.0364 | | -2.0175 | | -1.9903 | | -1.9607 | | -1.9363 | | -1.9085 | | -1.8873 | | -1.8623 | | -1.8369 | | -1.8113 | | -1.7805 | | -1.7589 | | -1.7299 | | -1.7089 | | -1.6851 | | -1.6595 | | -1.6366 | | -1.6068 | | -1.5906 | | -1.5729 | | -1.5575 | | -1.5410 | | -1.5264 | | -1.5069 | | -1.4909 | | -1.4770 | | -1.4623 | | -1.4530 | | -1.4393 | | -1.4242 | | -1.4124 | | -1.3959 | | -1.3807 | | -0.9642 | | -0.9653 | | -0.9638 | | -0.9676 | | -0.9690 | | -0.9703 | | -0.9695 | | -0.9734 | | -0.9722 | | -0.9751 | | -0.9756 | | -0.9793 | | -0.9820 | | -0.9810 | | -0.9868 | | -0.9905 | | | -6.8692 | | --- | | -6.4964 | | -6.1495 | | -5.8567 | | -5.5892 | | -5.3213 | | -5.1021 | | -4.8815 | | -4.6827 | | -4.4848 | | -4.3164 | | -4.1469 | | -3.9782 | | -3.8311 | | -3.6886 | | -3.5595 | | -3.4404 | | -3.3118 | | -3.1919 | | -3.0891 | | -2.9826 | | -2.8775 | | -2.7812 | | -2.6866 | | -2.5942 | | -2.5003 | | -2.4177 | | -2.3392 | | -2.2602 | | -2.1826 | | -2.5775 | | -2.5235 | | -2.4821 | | -2.4435 | | -2.4007 | | -2.3556 | | -2.3054 | | -2.2660 | | -2.2272 | | -2.1778 | | -2.1434 | | -2.0997 | | -2.0555 | | -2.0152 | | -1.9725 | | -1.9332 | | -1.8984 | | -1.8540 | | -1.8110 | | -1.7794 | | -1.7417 | | -1.7036 | | -1.6643 | | -1.6308 | | -1.5945 | | -1.5558 | | -1.5218 | | -1.4874 | | -1.4520 | | -1.4116 | | -1.3804 | | | -6.5991 | | --- | | -6.5408 | | -6.4740 | | -6.3899 | | -6.3248 | | -6.2480 | | -6.1625 | | -6.0811 | | -5.9823 | | -5.9120 | | -5.8259 | | -5.7351 | | -5.6427 | | -5.5400 | | -5.4303 | | -5.3325 | | -5.2194 | | -5.1004 | | -4.9781 | | -4.8547 | | -4.7217 | | -4.5872 | | -4.4450 | | -4.3068 | | -4.1673 | | -4.0060 | | -3.8735 | | -3.7129 | | -3.5753 | | -3.4224 | | -3.2875 | | -3.1520 | | -3.0241 | | -2.8901 | | -2.7670 | | -2.6431 | | -2.5277 | | -2.4092 | | -2.3066 | | -2.2028 | | -2.0931 | | -2.0092 | | -1.9167 | | -1.8267 | | -1.7531 | | -1.6748 | | -1.6012 | | -1.5302 | | -1.4640 | | -1.4266 | | -1.3926 | | -1.3587 | | -1.3306 | | -1.3019 | | -1.2712 | | -1.2456 | | -1.2139 | | -1.1920 | | -1.1683 | | -1.1394 | | -1.1206 | |


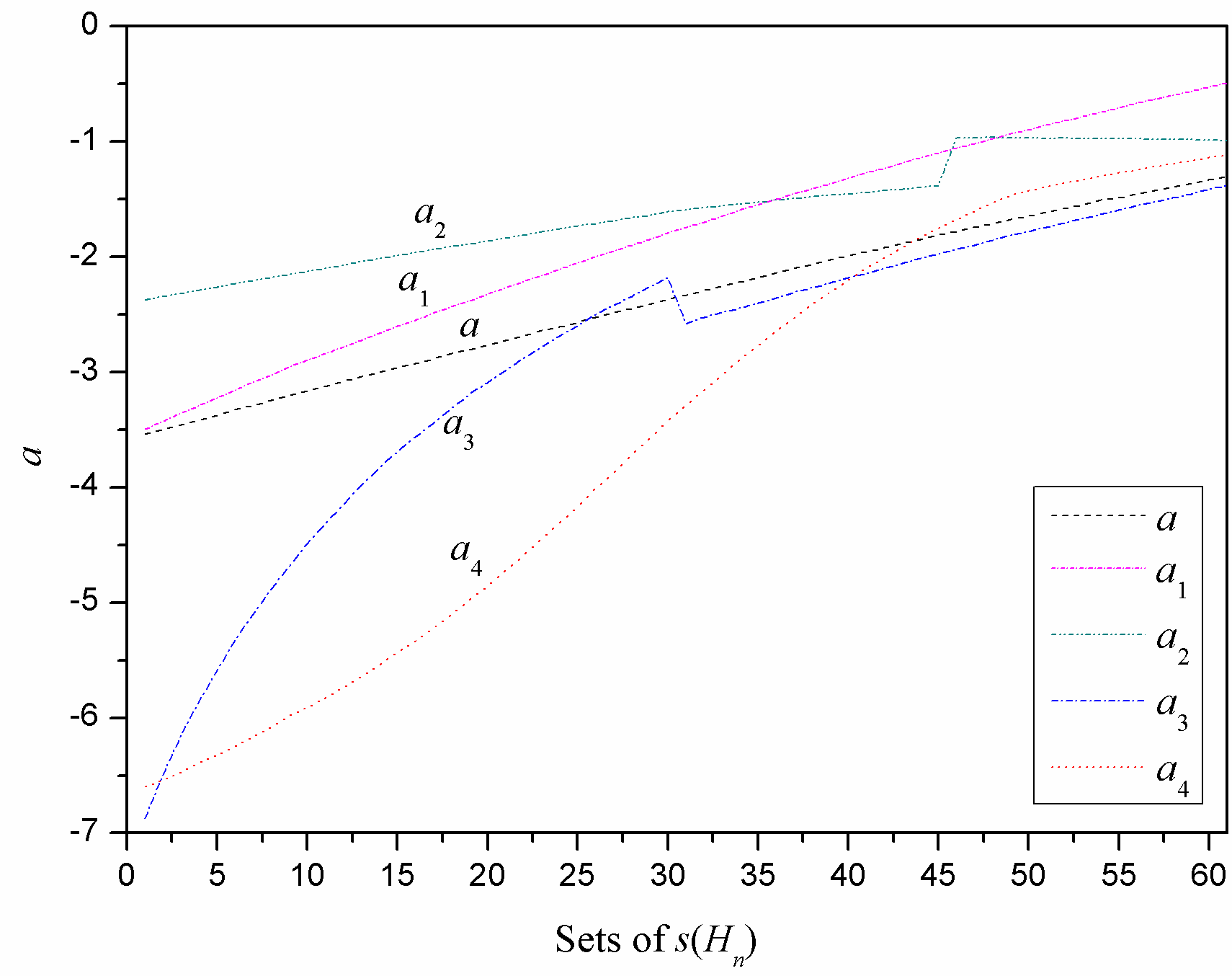


**Figure C.** Movement of the parameter *a* for decision makers and the group with the variation in $s(H_{n})$.

**Table F.** Values of the parameter *b* for decision makers and the group with the variation in $s(H_{n})$.

| ***λ*** | ***b*** | ***b*_1_** | ***b*_2_** | ***b*_3_** | ***b*_4_** |
| --- | --- | --- | --- | --- | --- |
| | 0.15 | | --- | | 0.145 | | 0.14 | | 0.135 | | 0.13 | | 0.125 | | 0.12 | | 0.115 | | 0.11 | | 0.105 | | 0.1 | | 0.095 | | 0.09 | | 0.085 | | 0.08 | | 0.075 | | 0.07 | | 0.065 | | 0.06 | | 0.055 | | 0.05 | | 0.045 | | 0.04 | | 0.035 | | 0.03 | | 0.025 | | 0.02 | | 0.015 | | 0.01 | | 0.005 | | 0 | | -0.005 | | -0.01 | | -0.015 | | -0.02 | | -0.025 | | -0.03 | | -0.035 | | -0.04 | | -0.045 | | -0.05 | | -0.055 | | -0.06 | | -0.065 | | -0.07 | | -0.075 | | -0.08 | | -0.085 | | -0.09 | | -0.095 | | -0.1 | | -0.105 | | -0.11 | | -0.115 | | -0.12 | | -0.125 | | -0.13 | | -0.135 | | -0.14 | | -0.145 | | -0.15 | | | 0.3353 | | --- | | 0.3370 | | 0.3372 | | 0.3385 | | 0.3398 | | 0.3414 | | 0.3422 | | 0.3435 | | 0.3457 | | 0.3466 | | 0.3483 | | 0.3493 | | 0.3513 | | 0.3521 | | 0.3533 | | 0.3547 | | 0.3553 | | 0.3573 | | 0.3590 | | 0.3602 | | 0.3614 | | 0.3635 | | 0.3638 | | 0.3661 | | 0.3677 | | 0.3683 | | 0.3697 | | 0.3711 | | 0.3726 | | 0.3732 | | 0.3746 | | 0.3766 | | 0.3779 | | 0.3801 | | 0.3808 | | 0.3814 | | 0.3828 | | 0.3851 | | 0.3862 | | 0.3878 | | 0.3890 | | 0.3909 | | 0.3921 | | 0.3932 | | 0.3945 | | 0.3960 | | 0.3973 | | 0.3983 | | 0.4003 | | 0.4015 | | 0.4025 | | 0.4046 | | 0.4060 | | 0.4070 | | 0.4084 | | 0.4099 | | 0.4113 | | 0.4129 | | 0.4143 | | 0.4154 | | 0.4168 | | | 0.9589 | | --- | | 0.9574 | | 0.9557 | | 0.9539 | | 0.9514 | | 0.9506 | | 0.9491 | | 0.9474 | | 0.9457 | | 0.9441 | | 0.9424 | | 0.9407 | | 0.9397 | | 0.9373 | | 0.9356 | | 0.9339 | | 0.9319 | | 0.9316 | | 0.9288 | | 0.9270 | | 0.9254 | | 0.9237 | | 0.9219 | | 0.9202 | | 0.9184 | | 0.9167 | | 0.9150 | | 0.9126 | | 0.9115 | | 0.9097 | | 0.9079 | | 0.9062 | | 0.9045 | | 0.9022 | | 0.9009 | | 0.8992 | | 0.8973 | | 0.8956 | | 0.8933 | | 0.8920 | | 0.8903 | | 0.8879 | | 0.8866 | | 0.8849 | | 0.8829 | | 0.8812 | | 0.8793 | | 0.8775 | | 0.8758 | | 0.8740 | | 0.8721 | | 0.8703 | | 0.8685 | | 0.8666 | | 0.8647 | | 0.8629 | | 0.8610 | | 0.8593 | | 0.8575 | | 0.8555 | | 0.8536 | | | 0.0915 | | --- | | 0.0939 | | 0.0979 | | 0.1028 | | 0.1049 | | 0.1082 | | 0.1132 | | 0.1164 | | 0.1184 | | 0.1210 | | 0.1251 | | 0.1296 | | 0.1310 | | 0.1332 | | 0.1377 | | 0.1409 | | 0.1440 | | 0.1471 | | 0.1512 | | 0.1541 | | 0.1570 | | 0.1607 | | 0.1634 | | 0.1659 | | 0.1704 | | 0.1750 | | 0.1790 | | 0.1806 | | 0.1838 | | 0.1870 | | 0.1907 | | 0.1943 | | 0.1980 | | 0.2022 | | 0.2056 | | 0.2067 | | 0.2101 | | 0.2118 | | 0.2145 | | 0.2197 | | 0.2235 | | 0.2269 | | 0.2272 | | 0.2322 | | 0.2367 | | 0.2388 | | 0.2407 | | 0.2443 | | 0.2475 | | 0.2522 | | 0.2538 | | 0.2575 | | 0.2607 | | 0.2639 | | 0.2671 | | 0.2705 | | 0.2736 | | 0.2759 | | 0.2790 | | 0.2822 | | 0.2852 | | | 0.3260 | | --- | | 0.3293 | | 0.3314 | | 0.3348 | | 0.3371 | | 0.3404 | | 0.3432 | | 0.3454 | | 0.3482 | | 0.3507 | | 0.3537 | | 0.3569 | | 0.3593 | | 0.3623 | | 0.3653 | | 0.3682 | | 0.3708 | | 0.3737 | | 0.3762 | | 0.3789 | | 0.3821 | | 0.3850 | | 0.3883 | | 0.3908 | | 0.3946 | | 0.3969 | | 0.4003 | | 0.4035 | | 0.4064 | | 0.4085 | | 0.4121 | | 0.4157 | | 0.4185 | | 0.4218 | | 0.4248 | | 0.4280 | | 0.4305 | | 0.4343 | | 0.4374 | | 0.4406 | | 0.4439 | | 0.4468 | | 0.4496 | | 0.4532 | | 0.4564 | | 0.4596 | | 0.4627 | | 0.4652 | | 0.4690 | | 0.4721 | | 0.4754 | | 0.4785 | | 0.4819 | | 0.4848 | | 0.4876 | | 0.4909 | | 0.4942 | | 0.4978 | | 0.5010 | | 0.5040 | | 0.5067 | | | 0.2329 | | --- | | 0.2332 | | 0.2329 | | 0.2337 | | 0.2346 | | 0.2346 | | 0.2349 | | 0.2360 | | 0.2357 | | 0.2361 | | 0.2367 | | 0.2373 | | 0.2372 | | 0.2387 | | 0.2380 | | 0.2384 | | 0.2394 | | 0.2393 | | 0.2402 | | 0.2403 | | 0.2411 | | 0.2415 | | 0.2426 | | 0.2434 | | 0.2439 | | 0.2424 | | 0.2436 | | 0.2445 | | 0.2448 | | 0.2454 | | 0.2464 | | 0.2456 | | 0.2469 | | 0.2479 | | 0.2482 | | 0.2489 | | 0.2500 | | 0.2514 | | 0.2495 | | 0.2516 | | 0.2524 | | 0.2530 | | 0.2535 | | 0.2542 | | 0.2549 | | 0.2555 | | 0.2566 | | 0.2570 | | 0.2579 | | 0.2597 | | 0.2595 | | 0.2605 | | 0.2612 | | 0.2605 | | 0.2623 | | 0.2634 | | 0.2640 | | 0.2653 | | 0.2661 | | 0.2655 | | 0.2674 | |


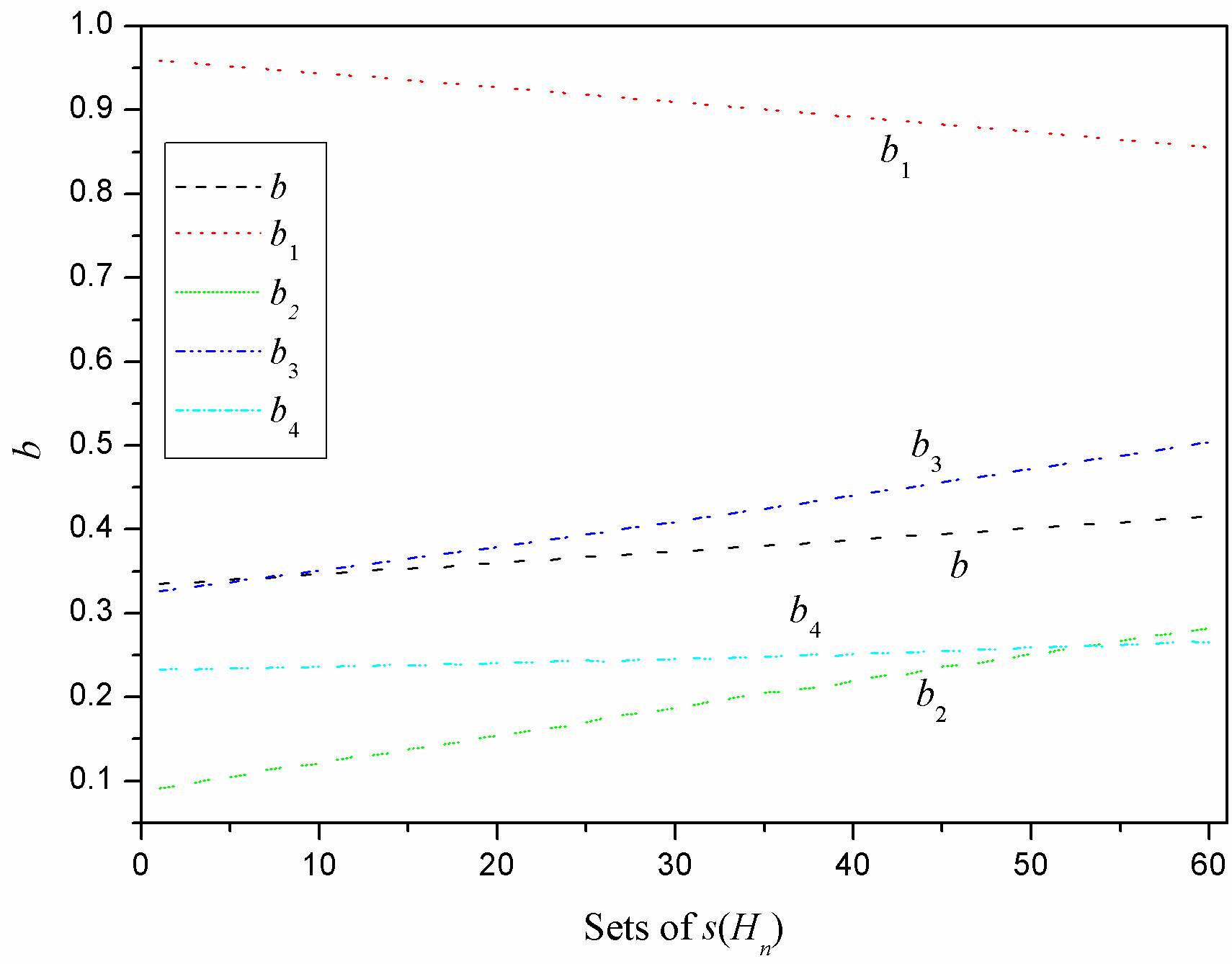


**Figure D.** Movement of the parameter *b* for decision makers and the group with the variation in $s(H_{n})$.
